# Developmental Changes in Nonsymbolic and Symbolic Fractions Processing: A Cross-Sectional fMRI Study

**DOI:** 10.1111/desc.70042

**Published:** 2025-09

**Authors:** Yunji Park, Priya B. Kalra, Yun-Shiuan Chuang, John V. Binzak, Percival G. Matthews, Edward M. Hubbard

**Affiliations:** 1 Department of Educational Psychology, University of Wisconsin – Madison, Wisconsin, USA; 2 Department of Psychiatry & Behavioral Sciences, Stanford University, California, USA; 3 Western Institute for Neuroscience, Western University, Ontario, Canada; 4 Department of Psychology, University of Wisconsin – Madison, Wisconsin, USA

**Keywords:** neural distance effects, neuronal recycling hypothesis, neural representational similarity analysis, ratio processing system, symbolic fraction acquisition

## Abstract

A substantial body of research has demonstrated that human and nonhuman animals have perceptually-based abilities to process magnitudes of nonsymbolic ratios (e.g., ratios composed by juxtaposing two-line segments). In prior work, we have extended the neuronal recycling hypothesis to include neurocognitive architectures for nonsymbolic ratio processing, proposing that these systems might support symbolic fractions acquisition. We tested two key propositions: (1) children should show neural sensitivity to nonsymbolic fractions before receiving formal fractions instruction, and (2) they should leverage this foundation by recruiting neural architectures for nonsymbolic fractions processing for symbolic fractions. We compared nonsymbolic and symbolic fractions processing among 2nd-graders (n = 28, ages 7.5–8.8), who had not yet received formal symbolic fractions instruction, and 5th-graders (*n* = 33, ages 10.3–11.9), who had. During fMRI scanning, children performed ratio comparison tasks, determining which of two nonsymbolic or symbolic fractions was larger. Both cohorts showed behavioral and neural evidence of processing nonsymbolic and symbolic fractions magnitudes, with performance modulated by numerical distance between stimuli. Consistent with our predictions, 2nd-graders recruited a right parietal-frontal network for nonsymbolic fractions but not for symbolic fractions, whereas 5th-graders recruited a bilateral parietal-frontal network for both, overlapping with but extending beyond that of 2nd-graders. Furthermore, nonsymbolic-symbolic neural similarity in the intraparietal sulcus was greater for 5th-graders than for 2nd-graders. These results present the first developmental neuroimaging evidence that neural substrates for nonsymbolic ratios exist before formal learning, which may be recycled to process symbolic fractions.

## 1 Introduction

Basic competence with symbolic numbers is a foundational knowledge set, required for everything from purchasing groceries, to calculating monthly expenses, to investing. Much like reading, this competence is so critical that societies world-wide strive to educate people so that it operates fluidly. At the same time, it is such an evolutionarily recent invention that it is difficult to imagine how human brains could have evolved specifically to process symbolic numbers. Dehaene and Cohen’s (2007) *neuronal recycling hypothesis* offers a plausible account for how such recent cultural inventions might be supported by recycling ancient primitive cognitive architectures whose functionality has been exapted to support formal learning (Dehaene and Cohen 2007).

For example, several theorists have argued that knowledge of whole number symbols can be grounded on neurocognitive systems dedicated to individuating and processing sets of non-symbolic elements or *numerosities*. These accounts suggest that this phylogenetically ancient *approximate number system* (or ANS) can be reoriented in development to support processing of modern number symbols (e.g., Gallistel and Gelman 2000; Piazza 2010). However, these ANS accounts have been largely limited to whole number learning. Indeed, theorists like Dehaene (2011) have proposed that the ANS—because it operates on nonsymbolic analogs to whole numbers—may be ill-suited for supporting other types of numbers such as fractions or decimals (e.g., Dehaene 2011; Feigenson et al. 2004; Gallistel and Gelman 2000; Matthews et al. 2016; but see Chesney and Matthews 2018; Clarke and Beck 2021 for notable exceptions that cast the ANS as compatible with processing rational numbers). Accordingly, Dehaene concluded that fractions “... defy intuition because they do not correspond to any preexisting category in our brain” (p. 76).

In contrast to the putative limitations of the ANS, a recent body of research has detailed a perceptual ability to process nonsymbolic fraction magnitudes, a type of nonsymbolic analog to symbolic fractions (e.g., Bhatia et al. 2020, 2022; Jacob and Nieder 2009b; Jacob et al. 2012; Lewis et al. 2016; Matthews et al. 2016; Meng et al. 2019; Starling-Alves et al. 2022). Investigations centering this nonsymbolic ratio (henceforth called nonsymbolic fractions for simplicity) processing ability stand to expand the neuronal recycling hypothesis to the realm of fractions (Jacob et al. 2012; Lewis et al. 2016; Matthews et al. 2016; Sidney et al. 2017).

Lewis et al. (2016) outlined a cogent account of how the neuronal recycling hypothesis might apply to cognitive architectures used for nonsymbolic fraction processing and how they might be used to build skills with fractions symbols. Here we tested two key propositions of this account: (1) that children should show substantial neural sensitivity to nonsymbolic fractions prior to receiving formal schooling on fractions and (2) that children should leverage this non-symbolic foundation by recruiting non-symbolic fractions processing architectures to process symbolic fractions.

### 1.1 | A Cognitive Primitive for Symbolic Fractions?

Converging lines of research have recently proposed that there is a primitive ability to process nonsymbolic fraction magnitudes, such as ratios instantiated by juxtaposing two line segments (e.g., Bonn and Cantlon 2017; Jacob et al. 2012; Matthews and Chesney 2015; Matthews et al. 2016). There are currently several competing accounts about the nature of the neurocognitive architectures that support nonsymbolic fraction processing. For instance, Jacob and Nieder (2009a, 2009b) proposed that the same nonverbal code used for absolute number might also encode ratio magnitudes (see also Chen and Verguts 2017; Clarke and Beck 2021). In contrast, others have posited that nonsymbolic fraction magnitudes are processed via a ratio processing system (or RPS), an ancient system sensitive to rational number magnitudes that is separate from the ANS (Lewis et al. 2016; Park et al. 2021). Still other accounts have proposed that there is a generalized magnitude system that compares individual absolute magnitudes that functions by mapping absolute magnitudes to an internal ratio scale (Bonn and Cantlon 2017; Chesney and Matthews 2018; but see Balci and Gallistel 2006). Despite their differences, what these accounts hold in common is the contention that there is an evolutionarily ancient, perceptually-based sensitivity to nonsymbolic fraction magnitudes. Thus, each is compatible with the idea that this ancient system might be “recycled” to support understanding of symbolic fractions and related concepts.

To date, the nonsymbolic fraction recycling account has been supported by several behavioral studies conducted with different age ranges. One line of research has shown indirect evidence for co-processing of nonsymbolic and symbolic fractions by using tasks indicating rapid, cross-format mapping of ratio magnitudes (e.g., Kalra et al. 2020; Matthews and Chesney 2015). Kalra et al. (2020) conducted cross-format fraction comparison tasks, whereby 2nd- and 5th-grade children compared which of two ratios (i.e., line ratios vs. symbolic fractions) were larger. In both grades, they observed a classic *distance effect* in which performance improved as the distance between ratios increased (e.g., Buckley and Gillman 1974). These cross-format distance effects showed that children can process both nonsymbolic and symbolic ratios as analog magnitudes. These cross-format distance effects parallel those found among adult populations (Binzak et al. 2019; Matthews and Chesney 2015). Additionally, reaction times in these experiments were rapid enough that the authors suggested that participants may compare nonsymbolic line ratios to symbolic fractions without first converting nonsymbolic stimuli to symbolic form. These consistent findings across children and adults suggest the possibility that nonsymbolic and symbolic fractions may be compared through a shared system, consistent with the recycling account.

Additional findings suggest that more precise acuity for discriminating nonsymbolic fraction magnitudes is correlated with increased competence with fractions and other symbolic mathematics domains among both children and adults (e.g., Begolli et al. 2020; Hansen et al. 2015; Matthews et al. 2016; Möhring et al. 2016; Park and Matthews 2021; Wong 2019). For instance, previous studies revealed that children’s performance on nonsymbolic proportion match-to-sample tasks predicted their fractions knowledge (e.g., Hansen et al. 2015; Möhring et al. 2016; Wong 2019; but see Bhatia et al. 2020). Similar studies with adults found nonsymbolic fraction processing ability predicts both fractions knowledge and algebra performance (Matthews et al. 2016; Park and Matthews 2021) even when controlling for domain general skills such as inhibitory control. These findings imply a potential link between nonsymbolic and symbolic fraction processing, consistent with the claim that nonsymbolic fraction processing serves as a cognitive primitive that is recycled for supporting symbolic fraction knowledge.

### 1.2 | Neural Substrates for Processing Ratio Magnitudes

Neuroimaging studies have also found that the same neural substrates may be used to support both nonsymbolic and symbolic representations of fraction magnitudes (Ischebeck et al. 2009; Jacob and Nieder 2009a, 2009b; Mock et al. 2018; but see Bhatia et al. 2022). For example, Jacob and Nieder (2009b) used a functional MRI adaptation paradigm to investigate the neural distance effect for nonsymbolic fractions. They observed that both the intraparietal sulcus (IPS) and prefrontal cortex (PFC) regions exhibited distance effects for nonsymbolic fraction magnitudes (represented by line and dot ratios). Notably, these regions have often been implicated in analog processing of numerical magnitudes (Dehaene et al. 1998; Nieder and Dehaene 2009; Piazza et al. 2004; Sokolowski et al. 2017). Moreover, Ischebeck et al. (2009) observed similar activation of the prefrontal-parietal network during symbolic fraction comparisons in adults. These parallel findings implicate the use of common neural substrates for processing both nonsymbolic and symbolic fraction magnitudes.

Building on these findings from between-subject paradigms, a few recent studies have employed within-subjects designs among adult participants and reported somewhat mixed results (Bhatia et al. 2022; Binzak 2020; Mock et al. 2018). Mock (2020) and Binzak (2020) used magnitude comparison paradigms in which participants compared fraction magnitudes in symbolic and nonsymbolic formats. Results revealed overlapping regions of neural activation, regardless of whether comparisons were made within or across nonsymbolic and symbolic formats. Task-specific activation was particularly apparent in the right inferior parietal lobules (including the IPS), and right prefrontal regions. Moreover, activation for cross-notation comparisons was characterized by neural distance effects, suggesting shared magnitude-dependent processing of nonsymbolic and symbolic representations at the neural level. These results further support the nonsymbolic fraction recycling hypothesis by demonstrating use of common neural substrates for nonsymbolic and symbolic within individuals (Rosenberg-Lee 2021; Wortha et al. 2022).

Beyond the regional overlap in averaged neural activations between nonsymbolic and symbolic fraction processing, a recent study employed both univariate and multivariate neural representational similarity analyses in adults (Bhatia et al. 2022). The authors used an adaptation task that sequentially presented four types of stimuli: individual nonsymbolic line segments, symbolic whole numbers, nonsymbolic line ratios, and symbolic fractions. Whereas univariate analysis identified significant adaptation effects for individual nonsymbolic lines and whole numbers, it failed to capture the same effects for nonsymbolic and symbolic fraction magnitudes. Furthermore, whole-brain multivariate analysis of neural representations in the IPS were largely explained by a format-specific (nonsymbolic vs. symbolic) model rather than a model accounting for numerical magnitudes. Based on these findings, Bhati et al. argued that the IPS does not process nonsymbolic and symbolic fractions as abstract magnitudes. Given the existence of such mixed results for potential IPS recruitment from studies using different fMRI paradigms, it is clear that more systematic research on the topic is still needed.

### 1.3 | Present Study

Although most recent studies have demonstrated that nonsymbolic and symbolic fractions are processed by similar substrates in adults, it is not clear *how* fractions in such dissimilar formats come to be processed by the same substrate during development. Indeed, studying adult participants exclusively cannot settle the question of whether a pre-existing cortical system for nonsymbolic fraction magnitude serves as the basis for symbolic fractions; answering this question requires a developmental approach. To our knowledge, there have been no neuroimaging studies to date investigating how children process nonsymbolic versus symbolic fractions at different developmental stages.

**FIGURE 1 |:**
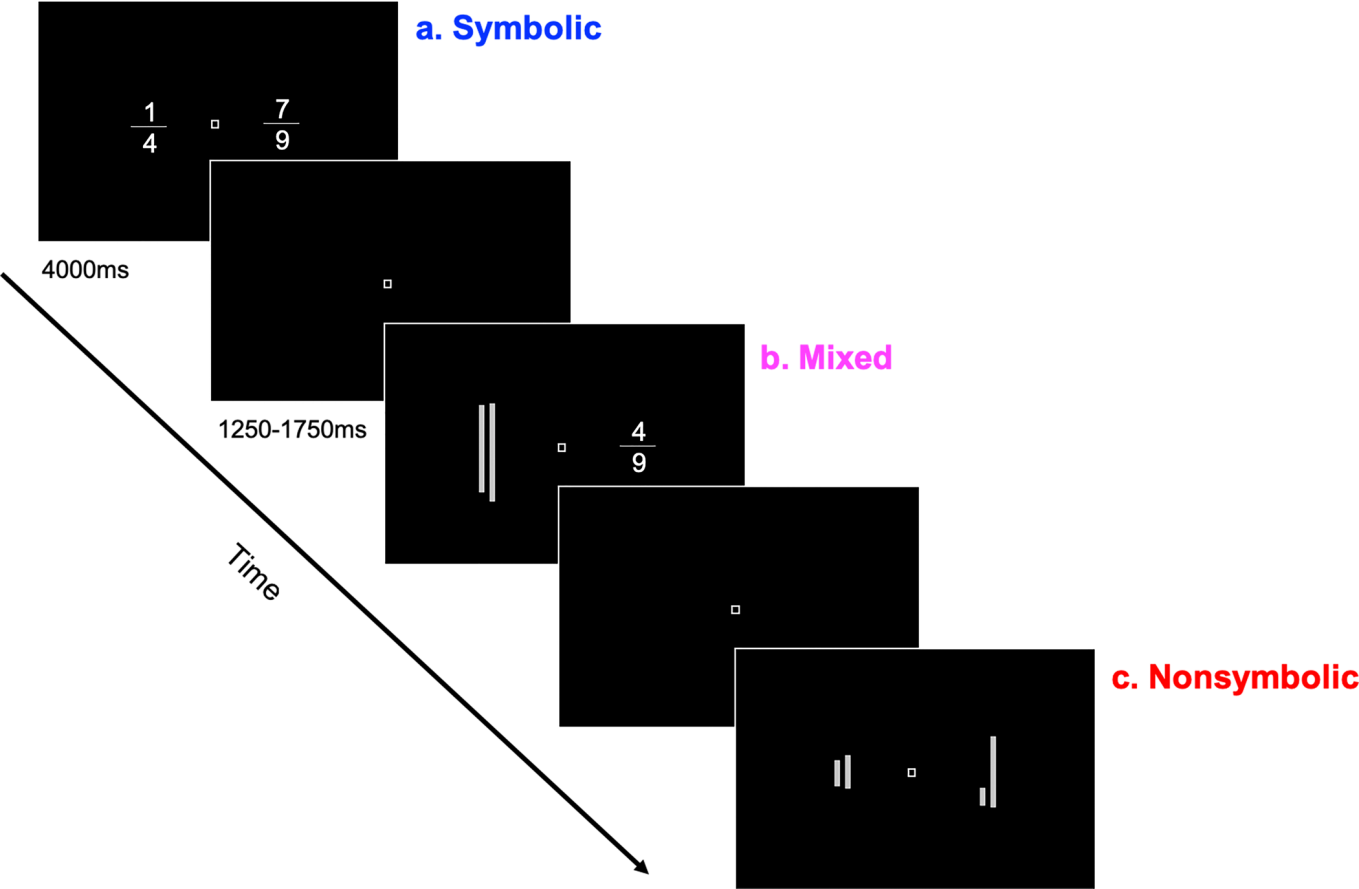
Sample trials from the comparison task. Trials type include (**A**) symbolic condition comparing symbolic fractions (Sym), (**B**) the mixed condition comparing symbolic fractions and line ratios (Mixed), and (**C**) the nonsymbolic condition comparing line ratios (Nonsym).

The aim of the current study was two-fold: (1) to explore similarities between nonsymbolic and symbolic fraction processing in school-aged children, and (2) to explore developmental differences in nonsymbolic and symbolic fraction processing during early years of fraction instruction. To this aim, we compared the behavioral and neural signatures of children who have not yet received formal fractions instruction (2nd-graders) and those of children who have received a few years of fractions instruction (5th-graders). Building on Kalra et al. (2020), we leveraged an extended fraction comparison task to investigate potential shared neural systems for processing nonsymbolic and symbolic fractions. In a scanner, participants completed comparisons in three different formats: (1) nonsymbolic notations comparing two lineratios, (2) mixed notations comparing symbolic and nonsymbolic fraction formats, and (3) symbolic notations comparing symbolic fractions (Figure 1).

Importantly, in addition to traditional univariate analysis, we employed multivariate neural representational similarity (NRS) analyses on *a priori*-defined regions of interest (ROIs) to examine whether children engage the IPS to process symbolic fractions in a similar manner as they do for nonsymbolic fractions. We specifically focused on the neural pattern *similarities* between the neural distance effects for nonsymbolic and symbolic fraction within the IPS in 2nd- and 5th-graders, similar to the previous studies investigating nonsymbolic and symbolic whole number mapping (Park, Zhang, Chang et al. 2024; Park, Zhang, Schwartz et al. 2024; Schwartz et al. 2021).

Per Kalra et al. (2020), we predicted that both 2nd- and 5th-grade children should behaviorally be able to compare both nonsymbolic and symbolic fractions. We additionally expected that nonsymbolic fraction comparisons would be the easiest and that symbolic fraction comparisons would be the hardest. Neurally, we expected that children in both 2nd- and 5th-grade would engage the same frontal and parietal brain regions, including the IPS, for nonsymbolic fraction processing as seen in adults (e.g., Ischebeck et al. 2009; Mock et al. 2018). We further hypothesized that among 5th-graders (1) both nonsymbolic and symbolic fraction processing would engage the IPS, and (2) neural patterns between two magnitude formats within the IPS would be significantly similar.

The second proposition of the recycling hypothesis is that if nonsymbolic fraction processing plays a role as a foundation for building symbolic fractions concepts, then symbolic fraction processing should begin to recruit similar neural patterns engaged in nonsymbolic fraction processing as children receive more instruction and practice with symbolic fractions. Thus, we predicted that 5th-graders, exposed to years of fractions instruction would exhibit greater similarities in neural patterns between nonsymbolic and symbolic comparisons in the IPS compared to 2nd-graders.

## 2 | Methods

### 2.1 | Participants

We recruited 47 second-grade (*M*_*Age*_ = 7.68, *SD*_*Age*_ = 0.43) and 45 fifth-grade (*M*_*Age*_ = 10.68, *SD*_*Age*_ = 0.47) children from public schools in and around Madison, Wisconsin as a part of a larger study of the development of children’s fractions abilities (See Kalra et al. 2020; Kalra, Hubbard, and Matthews 2020; Starling-Alves et al. 2022). All participants were right-handed native English-speakers with normal or corrected to normal vision (See Supporting Information for detailed demographic information). Parents or guardians gave written consent, and children gave verbal assent. All protocols were approved by the biomedical research ethics committee of the Institutional Review Board (2016–0665). Participants received monetary compensation and small gifts for their participation.

The students in our sample came from a number of different school districts, each of which used different curricula. However, all of the districts followed US Common Core standards (https://corestandards.org/), which indicate that direct systematic instruction with symbolic fractions should begin in 3rd grade. Additionally, the study team visited a number of these schools and spoke with educators in these districts, confirming that minimal formal (symbolic) fractions instruction occurs prior to 3rd grade. As part of the larger study from which the current fMRI sample was drawn, all participants completed a Fraction Knowledge Assessment composed of items measuring procedural and conceptual knowledge of fractions, drawn from several standardized assessments (see Kalra et al. 2020 for more details). In the larger behavioral sample, 2nd-graders were at or near floor (<10% correct) on all four fractions arithmetic problems. They also struggled to order three fractions by magnitude (10% correct) and had difficulties applying fractions labels and concepts to visual figures, such as shading a designated fraction of a figure (Shade ½, 50% correct) or drawing a line length in relation to another line (“draw a line ¼ as long as this line” or “draw a line three times as long as this line” 38% and 44% correct, respectively). Taken together, the analysis of the curricula and direct assessment of fractions skills demonstrated that the 2nd-graders in our sample had not received substantial formal instruction with symbolic fractions.

One 2nd-grade child was excluded due to an ADHD diagnosis, and two children (one 2nd-grade and one 5th-grade) failed to complete the scan due to excessive movement. Another two 5th-grade children were excluded due to technical issues during scan acquisition. We additionally excluded runs with head movement greater than 2.5 mm (see details in Section 2.6.2) and runs in which children showed chance level behavioral performance in the scanner. A participant’s full set of data were excluded if three or more out of the six functional runs were removed. Due to this filtering process, an additional seventeen 2nd-grade and nine 5th-grade children were excluded. After these exclusions, the final analytic sample consisted of 28 2nd-graders (*M*_*Age*_ = 7.68, *SD*_*Age*_ = 0.48) and 33 5th-graders (*M*_*Age*_ = 10.76, *SD*_*Age*_ = 0.44).

### 2.2 | Fraction Comparison Tasks

Participants completed fraction comparison tasks in the MRI scanner (Binzak 2020; Kalra et al. 2020) using stimuli presented by E-prime software (Psychology Software Tools, Shapsburg, PA). On each trial, participants compared two ratios made either from juxtaposed line segments forming nonsymbolic fractions or from symbolic fractions (Figure 1; see Table S1 for the list of the comparison stimuli). Comparison pairs were presented in each of three types: (1) Fractions versus Fractions (Symbolic, hereafter Sym), (2) Line ratios versus Fractions (hereafter, Mixed), and (3) Line ratios versus Line ratios (Nonsymbolic, hereafter Nonsym). Stimuli were presented side-by-side in a light gray on a black background. Participants selected the larger fraction by pressing the corresponding button with either their index (indicating the left fraction) or middle finger (indicating the right). Children completed at least one run of practice trials outside the scanner to become familiar with the task before the real scan.

To manipulate task difficulty, we varied the numerical distance between stimuli across trials, with numerical distance defined as the difference between the magnitudes of the compared fractions (|fraction A—fraction B|). Fraction pairs were organized into three distance bins for the purposes of analysis (Jacob and Nieder 2009b; Kalra et al. 2020): Near (0.048–0.233), Medium (0.262–0.446), and Far (0.514–0.750) (See Table 1). We predicted that participants would exhibit distance effects in both their neural and behavioral responses, consistent with findings in prior studies with fractions (e.g., DeWolf et al. 2016; Ischebeck et al. 2009; Mock et al. 2018). Specifically, we expected the Near condition to yield the lowest accuracy, the longest response times, and the greatest neural activation. In contrast, we expected that the Far condition should yield the highest accuracy, lowest response times, and least neural activation.

#### 2.2.1 | Stimuli

##### 2.2.1.1 | Symbolic Fractions.

We used the set of 27 irreducible proper fractions composed of single digit components. Building on our previous analysis of the impact of various controls on symbolic fraction comparison (see Binzak and Hubbard 2020), we selected 36 pairs from the 351 possible unique pairings with stimuli balanced across pairs. Following the nomenclature of Obersteiner et al. (2013) which systematically investigated the impact of the strategy based on different types of fraction pairs, we have labeled each pair as follows: (1) both shared a common denominator (C*ommon Components* C*ongruent* pairs; CC-C), (2) the numerically larger fraction had a larger numerator and a smaller denominator than the smaller fraction (*Without Common Componentas Neutral* pairs; WCC-N), (3) the numerically larger fraction had a larger numerator and a larger denominator (*Without Common Component Congruent* pairs; WCC-C), and (4) the larger fraction had a smaller numerator and denominator than the smaller fraction (*Without Common Components Incongruent* pairs; WCC-IC) (see Kalra et al. 2020; Obersteiner et al. 2013; Table S1). However, it should be noted that our comparison task does not include *Common Components Incongruent* pairs, as presented in Obersteiner et al. (2013). Furthermore, in the Far distance bin, it was impossible to include incongruent numerator pairs because no qualifying pair existed with distance greater than 0.306 given the set of irreducible fractions with single digit components.

##### 2.2.1.2 | Nonsymbolic Fractions.

Nonsymbolic fractions were composed of pairs of juxtaposed gray lines. To build the nonsymbolic fractions, we used our symbolic fraction pairs as the reference, keeping the same holistic fractional magnitudes and distances between pairs across notation conditions. To minimize the probability that participants used each line-length (i.e., the numerator or denominator component) as a cue to make the comparison decision, we created two sets of line ratios (see Table 2). One set (numerator controlled) was controlled to minimize the correlation between the numerator length and overall fraction magnitude. The numerator length was randomly generated between 33 and 336 pixels, and the corresponding denominator length was then determined. The other set (denominator controlled) was controlled to minimize the correlation between denominator length and overall fraction magnitude. The denominator length was randomly generated to be between 130–300 pixels, and corresponding numerator length was then determined.

#### 2.2.2 | Task Procedure

For functional data acquisition, we used an event-related design, in which each participant completed six runs of 36 trials per run for a total of 216 trials. Each run included an equal number of trials from each notation condition (Nonsym, Mixed, and Sym), and the trials in each notation were evenly distributed among the distance bins (Near, Medium, and Far). All notations and distances were counterbalanced across the six runs. The number and the order of stimulus types were counterbalanced across participants (e.g., denominator or numerator controlled for a line ratio; see Stimuli section for details). Within each run, stimulus presentation order was random across different notations and distances for each participant. Run order was counterbalanced across participants.

Each trial began with a fixation cross, presented for 1250–1750 ms (with the range corresponding to randomly selected jitter, 1500 ms ± 250 ms) followed by the presentation of fraction stimuli. Participants could respond upon stimulus onset, and stimuli remained on-screen until the trial timed out after 4000 ms even if participants failed to respond.

### 2.3 | Experimental Procedure

#### 2.3.1 | Participation in Behavioral Study

Children who participated in the current neuroimaging study were a part of a large cross-sequential study investigating the development of fraction ability among primary and middle school children (see Kalra et al. 2020). In this study, children underwent multiple behavioral sessions during which they completed various neuropsychological, cognitive, and behavioral assessments. These included a PowerPoint instruction session introducing the concept of nonsymbolic and symbolic fractions (see Section 2.3.2 below), followed by a shortened version of the fraction comparison task. The shortened task comprised half of the trials from the task used during the MRI scan session (Kalra et al. 2020). After completing the behavioral portions of the study, we screened a subset of participants for the fMRI session based on their behavioral performance on the comparison task (requiring above chance level for inclusion) and their ability to remain still during a mock scan, as assessed by trained research assistants in the lab. For example, a child showing excessive movement such as frequent foot motion was screened out. During the mock scan procedure, all participants completed 1–2 runs of the same fraction comparison task as in the real scans to get comfortable with performing the task in the scanner environment.

#### 2.3.2 | Fraction Instruction

Because US 2nd-graders typically have not yet received formal instructions on fractions, and because the nonsymbolic line ratios were an unfamiliar format, all children received a brief PowerPoint lesson^1^ (also used by Kalra et al. 2020) introducing the concept of ratios/fractions prior to experimental runs. To introduce nonsymbolic and symbolic fractions in a child-friendly manner, we used cartoon characters to depict how height comparisons can make a ratio. Children were instructed that, “Joey is half as tall as Sara. When we think of Sara’s and Joey’s heights together, we can call it a RATIO. And we use numbers to talk about ratios.” Cartoon characters were eventually replaced by lines, so that children could gain some familiarity with the types of line ratios that would be presented as stimuli. When we introduced line ratios, the corresponding symbolic fractions were also presented simultaneously (See further details in Kalra et al. 2020).

### 2.4 | Data Acquisition

Participants were scanned in a General Electric 3-Tesla scanner (GE Medical Systems, Waukesha, WI) equipped with a 32-channel array head coil (Nova Medical) at the University of Wisconsin–Madison. Foam padding was used to limit head motion. Structural images were collected by using motion-corrected 3D T1-weighted (T1w) MPnRAGE with 1 mm isotropic resolution (TR = 4.876 ms, TE = 1.82 ms, Flip angle = 4°, FOV = 224 mm × 224 mm, in plane resolution: 256 × 256 pixels, the number of axial slices = 176) (Kecskemeti et al. 2016). Functional images were acquired with a 3D T2-weighted (T2w) echo-planar imaging sequence (TR = 2000 ms, TE = 22 ms, Slice thickness = 3 mm, Flip angle = 75°, FOV = 224 mm × 224 mm, 128 × 128 matrix). Each volume consisted of 38 slices (1.75 × 1.75 × 3 mm voxel size) with a 52 ms inter slice interval. The first five volumes of each functional run, during which participants waited for the task to begin, were also collected to allow for T2 equilibration effects. In total, 110 volumes were acquired for each functional run.

### 2.5 | Behavioral Analysis: The Fraction Comparison Task

#### 2.5.1 | Behavioral Distance Effects

To examine behavioral distance effects, we conducted mixed-effects logistic and linear regressions, for error rates and reaction times, respectively, to account for within-subject correlation among trials. We used the ‘gImer’ and ‘lmer’ functions of the’ lme4’ package in R software for each analysis (Bates et al. 2015). The degrees of freedom were estimated using Satterthwaite’s approximation under the ‘lme4’ package (Kuznetsova et al. 2017).

For each logistic and linear mixed-effects regression model, we regressed error (incorrect: 0 or correct: (1) or reaction times against notation (three levels, Nonsym, Mixed, Sym), reversed absolute holistic distance (i.e., |fraction1 – fraction2| multiplied by negative one (i.e., −1), and grade (2 levels: 5th-graders = 0, 2nd-graders = 1), such that higher levels exhibit higher error rates and reaction times. To facilitate comparison with our neuroimaging analyses, which used binned distances, we also conducted a parallel analysis for both error rates and reaction times to using the same binned distances. We used a backward difference coding scheme to allow binary comparison of variables at different levels as specified by our hypotheses (i.e., larger distance < smaller distance, Far < Med < Near) based on prior behavioral investigation of the same tasks (Kalra et al. 2020). Interactions among levels were also examined. Similar mixed effects regression analyses were conducted using ordered binned distance (Far < Med < Near) to confirm findings from the analyses.

#### 2.5.2 | Control Analysis

Prior work has noted that participants sometimes use heuristic strategies for fraction comparisons (e.g., Fazio et al. 2016; Morales et al. 2020; Obersteiner et al. 2013; Schneider and Siegler 2010). For instance, children can employ several componential strategies—such as using numerator or denominator distance across stimuli—which often generate correct responses. One recently emphasized heuristic is the *gap* strategy (Gap = Denominator—Numerator), which often yields correct answers due to the fact that larger fractions often have a smaller difference between their numerators and denominators compared to smaller fractions (Morales et al. 2020). To account for potential confounding factors that may influence distance effects, particularly in symbolic fraction comparisons, we conducted several additional analyses regressing error rates or reaction times against: (1) numerator and denominator distances and (2) gap distances (|(fraction 1: denominator 1 − numerator 1) − (fraction 2: denominator 2 − numerator 2)| in symbolic fraction comparisons.

In addition to componential strategies, previous studies have suggested that other cognitive demands, such as inhibitory control (Abreu-Mendoza et al. 2023; Rosenberg-Lee 2021), or task switching (Monsell 2003; Schliephake et al. 2022), might influence magnitude comparison tasks. It is plausible that children may have performed better (a) on fraction comparisons when a larger fraction had a larger numerator compared with when it had the smaller numerator because the latter might require inhibition, or (b) when trials with the same notation were repeated compared to when notation changed between trials, which might be treated as a task switch. Given the prior studies demonstrated priming effects in fraction comparisons (Abreu-Mendoza et al. 2020; Rossi et al. 2019), we specifically focused on cases in which the notation of preceding trial differed. To account for these potential influences of cognitive demands on the observed distance effects, we tested additional mixed-effects regression models considering: (1) congruency of the stimuli (CC-C, WCC-C, WCC-N, and WCC-IN, see Stimuli section above) for symbolic fraction pairs, and (2) whether trials were presented with a different notation from the preceding trial (trials in which the notation is same as the preceding trial = 0; trials in which notation is different from the preceding trial = 1). Detailed methods are described in Supporting Information.

### 2.6 | Imaging Analysis

#### 2.6.1 | Preprocessing

Acquired images were preprocessed and analyzed using SPM12 (Ashburner et al. 2014). The first five volumes of each functional run were discarded to account for the T2 equilibration effects. After realignment and slice-timing correction, the preprocessed functional images were co-registered to the T1 anatomical images and then spatially normalized to the standard Montreal Neurological Institute (MNI) template space using affine transformation, similar to standard practice in neuroimaging research with children (Cantlon et al. 2006; Kersey and Cantlon 2017a; Nakai et al. 2023; Perrachione et al. 2016). The resulting transformation parameters were then applied to the functional images, which were then resampled to isotropic voxel-sizes of 2 mm.

#### 2.6.2 | Head Motion

To rule out runs with excessive movement, we excluded those exhibiting head motion exceeding 2.5 mm over each run, considering both rotational and translational movements. We also manually inspected the movement plot for each run to check for excessive spikes, but none were observed. We additionally checked for possible differences in head motion between grades. We regressed movement parameters against grade using fixed effects models. We found no differences between 2nd- and 5th-graders in terms of translational (*β* = −0.040, *t* = –0.404, *p* = 0.688) or rotational (*β* = −0.014, *t* = −0.747, *p* = 0.457) movements.

#### 2.6.3 | First-Level Statistical Analysis

Following standard preprocessing of functional data, task-related brain responses were analyzed using a general linear model (GLM) implemented in SPM 12. Brain responses associated with correct trials for each distance condition and notation (distances: Near, Medium, and Far; notations: Nonsym, Mixed, and Sym) were modeled using a boxcar function matching the trial duration and convolved with a canonical hemodynamic response function. Incorrect trials were modelled with an error regressor as a regressor of no interest. Additionally, six motion parameters (translational and rotational movements) were included as regressors of no interest. Serial correlations in the fMRI time series were addressed using a first-degree autoregressive model. The applied GLM generated voxel-wise contrast maps for each participant.

#### 2.6.4 | Univariate Whole-Brain Analyses

For univariate analysis, we conducted a three-way mixed-effects ANOVA with grade (2nd vs. 5th) as the between-subject factor, and distance (Near, Medium, Far) and notation (Nonsym, Mixed, Sym) as the within-subject factors. We first examined the main effect of grade to determine whether 2nd-and 5th-graders exhibited differences in functional activation during the task. Next, we examined the main effect of distance to identify the regions of the brain showing greater activity for near distances relative to far distances—indicative of neural distance effects (NDEs)—across grades. We focused on the Near versus Far distance conditions to effectively capture differences in functional activations related to ratio magnitudes. We also examined the Near versus Med and the Med versus Far conditions to confirm our hypothesis. Furthermore, to better characterize the NDEs for each grade cohort, we conducted whole-brain *t*-tests contrasting the Near versus Far while collapsing across different notations. We then conducted similar whole-brain *t*-tests separately within each notation to isolate notation-specific NDEs.

Finally, we examined the interaction effects between grade and distance to investigate developmental differences in fraction magnitude processing. We conducted a mixed-effects ANOVA to examine significant interactions between Distance (Near vs. Far) and Grade (2nd- and 5th-graders) across different notations.

All the whole brain contrasts were masked with a gray matter mask, and significant clusters were identified using a height threshold of *p* value of 0.005 (cf., Kersey et al. 2019; Matejko and Ansari 2019, 2021; Schwartz et al. 2021 with children). Multiple-comparisons were corrected using a family-wise error rate (FWE) threshold of *p* < 0.05, with a minimum cluster size of 67 voxels, determined through Monte Carlo simulations. Critically, this approach avoids false positive results due to invalid cluster inferences (Eklund et al. 2016). To confirm the robustness of the effects, identical whole brain analyses were also performed using a more stringent height threshold of *p* < 0.001, with multiple comparisons corrected using the FWE threshold of *p* < 0.05, with a minimum cluster size of 30 voxels (see Supporting Information). Figures with inflated brain images were generated by mapping thresholded *t*-maps using ‘nearest voxel’ algorithm implemented in BrainNet Viewer or Nilearn Python package. Figures displaying sliced brain images were generated using MRIcroGL.

#### 2.6.5 | Multivariate Analysis: Neural Representational Similarity Analysis

To rigorously test our recycling hypothesis, we examined the similarity in neural representations of fraction magnitudes across nonsymbolic and symbolic notations by computing the spatial correlation of multivariate brain activity patterns between NDEs observed in each notation. Unlike univariate approaches that focus on brain activation levels, this multivariate analysis provides a way to assess similarities in multivoxel activation patterns, offering insights into shared neural representations across different cognitive processes (Haxby et al. 2014; Kriegeskorte et al. 2008).

To determine whether the IPS processes nonsymbolic and symbolic fraction magnitudes similarly, we performed a neural representational similarity (NRS) analysis (Kriegeskorte et al. 2006) on a priori-defined regions of interest (ROI). We used the IPS coordinates from Sokolowski et al. (2017)’s meta-analysis of nonsymbolic and symbolic number processing. In particular, we used five locations (three in the right IPS and two in the left IPS) identified in the meta-analysis as regions involved in processing both nonsymbolic and symbolic numbers. The reported coordinates in the Talairach (TAL) space ([36, −46, 44], [38, −42, 42], [32, −46, 44], [−26, −54, 44], [−34, −48, 44]) were transformed into MNI space using a MATLAB script (tal2icbm_spm) provided by the icbm2tal transformation tool (Lancaster et al. 2007). Using the transformed coordinates, each ROI was defined as a spherical region with a 6-mm radius centered on each peak coordinate.

Within each spherical ROI, the similarity between the Near versus Far distance effects across nonsymbolic and symbolic notations was calculated by computing the spatial correlations of voxel-wise brain activation patterns. The resulting spatial correlation values were then Fisher z-transformed (NRS value), with each transformed value assigned to the center voxel of the region.

Using the extracted NRS values, we conducted a one sample *t*-test to determine whether the degree of similarity between nonsymbolic and symbolic fractions is significantly different from zero-correlation (NRS value = 0). Next, a two-sample *t*-test comparing 2nd- and 5th-graders was conducted to examine developmental differences. Cohen’s *d* was calculated to assess effect size.

## 3 | Results

### 3.1 Behavioral Analysis

Children in both grades were capable of accurately and rapidly choosing the larger of two fraction magnitude stimuli (Error rates: 2nd-graders: *M* = 0.09, *SD* = 0.03, range = 0.04–0.15; 5th-graders: *M* = 0.06, *SD* = 0.03, range = 0.01–0.14; Reaction times (ms): 2nd-graders: *M*_*rt*_ = 1618.85, *SD* = 220.37, range = 1056.95–1986.51; 5th-graders: *M*_*rt*_ = 1520.69, *SD* = 269.06, range = 987.86–2207.03) across all notations (see Table 3 for results disaggregated by notation).

To test for the presence of behavioral distance effects, we performed mixed-effects regression analyses for both error rates and reaction times.

#### 3.1.1 | Behavioral Distance Effects

##### 3.1.1.1 | Error Rates.

**FIGURE 2 |:**
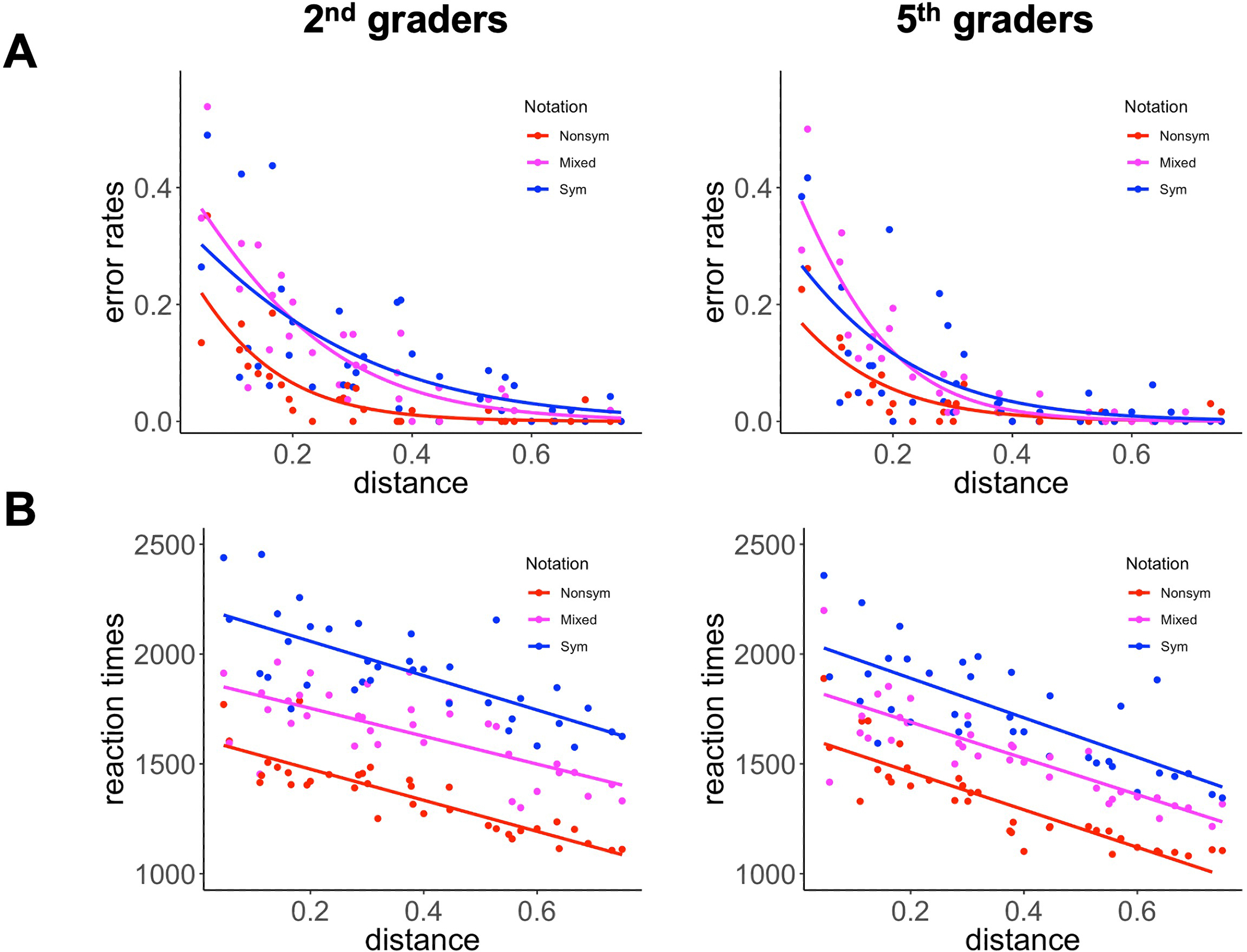
Displays of (**A**) error rates and (**B**) reaction times for 2nd-graders (left) and 5th-graders (right) by notation (Nonsym, Mixed, and Sym) and absolute distances. Each point indicates mean performance in each numerical distance across participants. Both graders exhibited significant distance effects such that error rates and reaction times increased as the distance between two fraction magnitude pairs decreased (*p* < 0.001), and significant notation effects (*ps* < 0.001) such that error rates and reaction times increased in the following order: nonsymbolic (Nonsym) (fastest), mixed (Mixed), symbolic (Sym).

We first regressed error rates against grade (5th- and 2nd-graders), notation (Nonsym, Mixed, Sym), and reversed absolute holistic distance (larger distance < smaller distance), such that the higher level exhibits higher error rates. As predicted, we found a significant effect of grade, whereby 2nd-graders were more likely to make errors than 5th-graders (*Odds Ratio* [*OR*] = 2.48, *p* < 0.001) (Table 4; Figure 2A). Critically, we found significant distance effects, as the likelihood of making an error on smaller distance trials was higher than for larger distance trials (*OR* = 1869.23, *p* < 0.001). As for notation effects, the likelihood of making an error on Mixed was higher than for Nonsym (*OR* = 2.48, *p* < 0.001). However, contrary to our predictions, the likelihood of making an error in Sym was lower than that of Mixed (*OR* = 0.59, *p* = 0.001) (Table 4). More importantly, we found a significant grade × distance interaction (*OR* = 0.24, *p* = 0.029). Subsequent analysis performed on each notation revealed that a significant grade × distance interactions was found in Mixed (*OR* = 0.04, *p* = 0.001) and Sym (*OR* = 0.14, *p* = 0.014), but not in Nonsym (*OR* = 2.71, *p* = 0.501). These results indicate that 5th-graders showed stronger distance effects for symbolic fractions compared to 2nd-graders.

##### 3.1.1.2 | Reaction Times.

We expected 5th-graders would be faster than 2nd-graders in parallel with error rate patterns. However, analysis showed that 5th-graders were not significantly faster than 2nd-graders (*β* = 0.04, *p* = 0.458), likely due to the similar reaction times for nonsymbolic fraction comparisons. For other latency effects, the results were consistent with the error rate analyses. We found a significant distance effect for reaction times, whereby participants responded slower on smaller distance trials than on larger ones (*β* = 0.25, *p* < 0.001) (Table 5; Figure 2B). Both grade cohorts responded faster for Nonsym than for Mixed notations (*β* = 0.20, *p* < 0.001), and responded faster for Mixed than for Sym notation (*β* = 0.18, *p* < 0.001) consistent with our hypothesis (Kalra et al. 2020). Furthermore, although we did not find grade effects, we found significant grade × notation interactions (*β* = 0.04, *p* = .025), and between grade × distance interactions (*β* = −0.05, *p* = 0.004). Subsequent analysis performed on each notation revealed that a significant grade × distance interactions in Mixed (*β* = −0.04, *p* = 0.030) and a marginally significant interaction in Nonsym (*β* = −0.06, *p* = 0.056), but no significant interaction in Sym (*OR* = −0.07, *p* = 0.208). These results suggest that in contrast to the error rate findings, 5th-graders showed stronger distance effects in reaction times for nonsymbolic fractions compared to 2nd-graders. To facilitate comparison with our neuroimaging analyses, we conducted a parallel behavioral analysis for both error rates and reaction times using binned distances, which revealed similar patterns of results as the analyses based on linear distances (Tables S2 and S3; Figure S1).

To summarize, we observed notation and distance effects across 2nd- and 5th-graders for both error rates and reaction times across all notations, as well as developmental differences in processing of different notations and distance effects between 2nd- and 5th-graders.

#### 3.1.2 | Behavioral Distance Effects and Potential Confounding Variables

Although we found significant distance effects, it is crucial to confirm that these effects were not driven by potentially confounding variables. In our previous behavioral study using this same paradigm and larger samples of 2nd- and 5th-graders (Kalra et al. 2020), control analyses demonstrated that participants relied on holistic distance when comparing fractions rather than heuristic strategies such as gap (denominator—numerator) comparisons, numerator distance, or benchmark comparisons. To verify this same reliance on holistic magnitude for the in-scanner behavioral data, we performed a set of additional logistic and linear mixed-effects regression analyses accounting for numerator and denominator distances, gap distances, and the presence of common components in symbolic fraction comparisons within the in-scanner behavioral data. Our results confirmed that holistic distance effects remained significant and robust (*ps* < 0.001) even after accounting for possible use of these componential strategies (See details in Supporting Information, Tables S4 and S5, and Figure S2). Moreover, the effects of holistic distance were much larger than those due to the alternative heuristics.

Furthermore, we examined the influence of congruency of the symbolic fraction stimuli (i.e., congruent < neutral < incongruent; See Methods and Table S1 for the stimuli information), which may be related to inhibitory control, as well as the costs for switching tasks across different notation conditions. Our results confirmed that distance effects, observed through error rates and reaction times, remained significant even after controlling for cognitive demands related to inhibitory control and notation switching (*ps* < 0.001). We also observed a significant effect of congruency on both error rates (*p* < 0.001) and reaction times (*p* = 0.003) along with a significant interaction with distance and reaction times (*p* = 0.004). In contrast, switching demands did not affect overall performance (*ps* > 0.645) or distance effects in either error rates or reaction times (*ps* > 0.078). (See Supporting Information, Tables S6 and S7 and Figure S3). Taken together, these control analyses confirmed significant behavioral distance effects among both 2nd- and 5th-grade children, even after accounting for the potential use of alternative strategies and the possible impact of extraneous cognitive demands.

### 3.2 | Neuroimaging Analysis

#### 3.2.1 | Developmental Differences Between 2nd Versus 5th Graders

**FIGURE 3 |:**
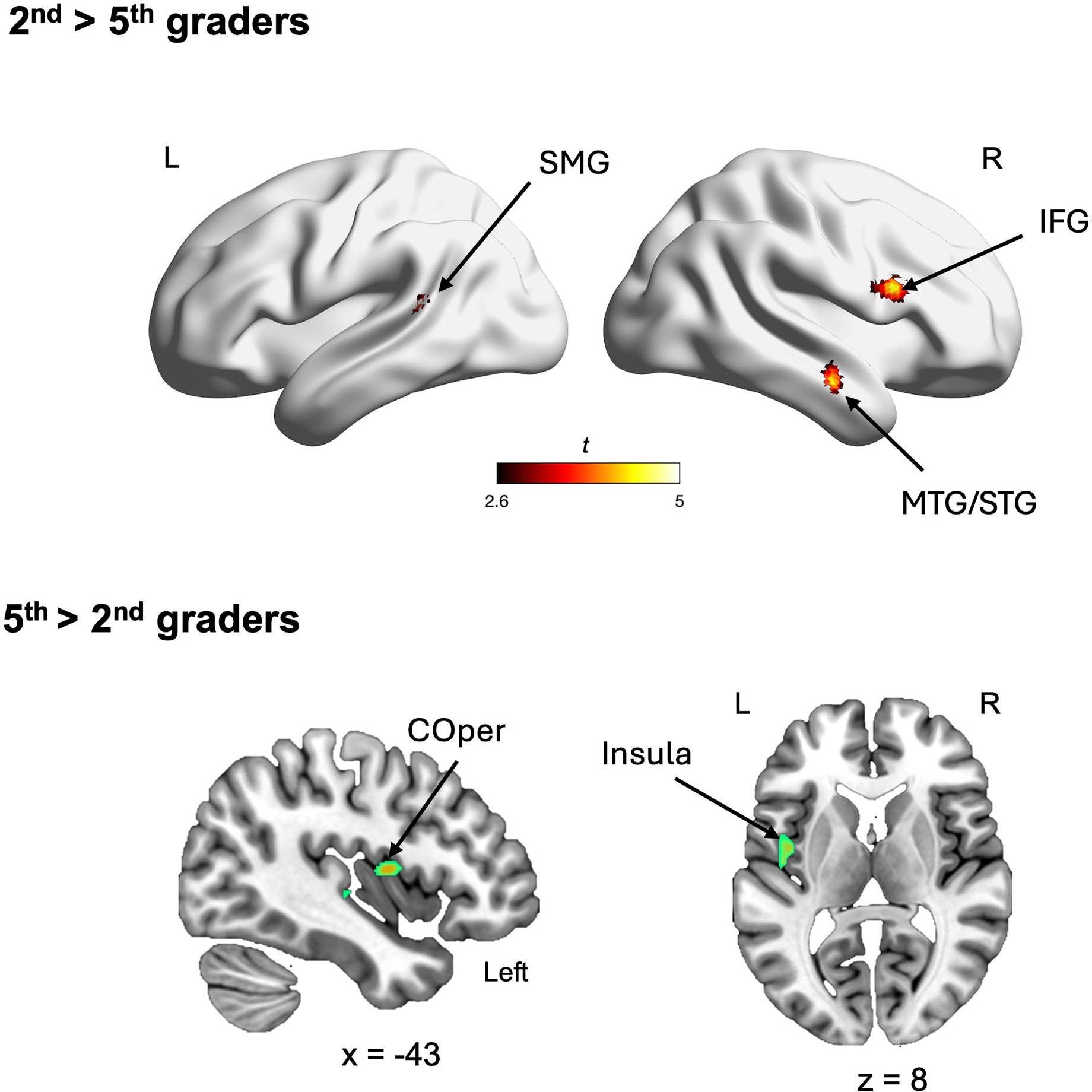
Significant main effect of grade identified by whole brain mixed-design ANOVA with Grade (2nd- and 5th-graders), Notation (Nonsym, Mixed, Sym) and Distance (Near, Medium, Far). Specifically, 2nd-graders exhibited greater activations in the distributed brain regions, including the left supramarginal gyrus (SMG), a cluster spanning the right middle and superior temporal gyrus (MTG and STG), and the right inferior frontal gyrus (IFG) (**Top**). In contrast, 5th-graders exhibited greater functional activations in a single cluster spanning the left central operculum (COper) and insula (**Bottom**). L, Left; R, Right. The color bar represents *t*-values.

To examine the brain regions exhibiting task-related activation differences between 2nd- and 5th-graders, we conducted a whole-brain mixed-effects ANOVA with grade (2nd- and 5th-grades) as a between-subjects factor, and distance (Near, Medium, Far) and notation (Nonsym, Mixed, Sym) as within-subject factors. The ANOVA analysis revealed a significant main effect of grade in both directions. Specifically, 2nd-graders exhibited greater task-related activations (Task > Baseline) across multiple regions, including the left supramarginal gyrus (SMG), a cluster spanning the right middle and superior temporal gyrus (MTG and STG), and the right inferior frontal gyrus (IFG). In contrast, 5th-graders exhibited greater functional activations in a single cluster spanning the left central operculum (COper) and insula (Figure 3; see Table S8). Notably, greater activation observed in 2nd-graders compared to 5th-graders remained significant even with a more stringent height threshold of uncorrected *p* < 0.001, whereas the reverse pattern did not (See Figure S4). These results indicate that 2nd-graders exhibited greater activations during the task compared to 5th-graders.

#### 3.2.2 | Neural Distance Effects Across All Notations in 2nd- and 5th-Graders

**FIGURE 4 |:**
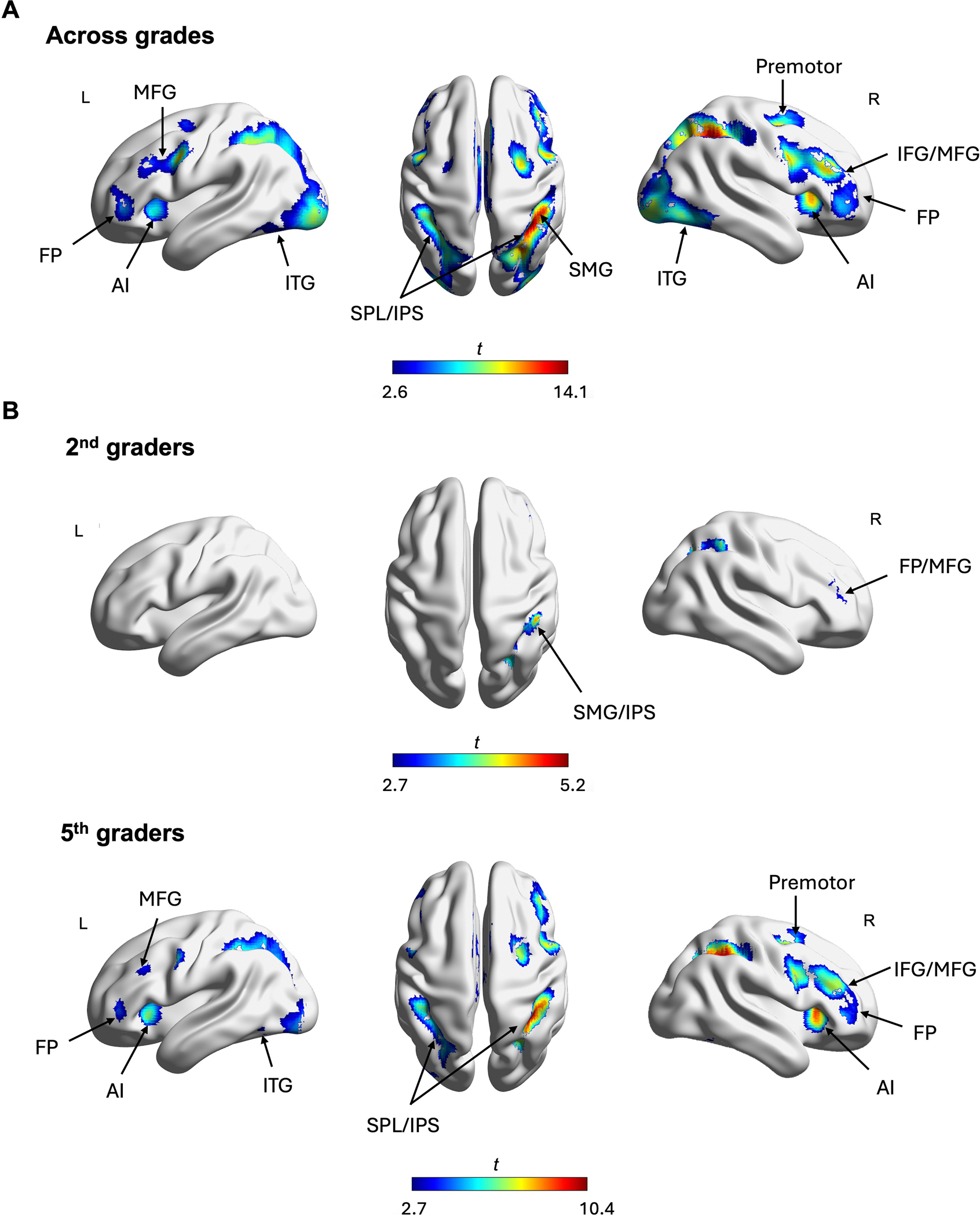
Significant distance effects (Near vs. Far) controlling for notations (Nonsym, Mixed, Sym) across grades and in each grade. **(A)** The whole brain mixed-design ANCOVA revealed the main effect of distance (Near, Med, Far). The largest neural distance effects (NDE) across all notations and grades were identified between the Near versus Far contrasts (see Supporting Information for the Near vs. Med and Med vs. Far contrasts) in multiple brain regions including the bilateral superior parietal lobule (SPL), inferior parietal sulcus (IPS), inferior and middle frontal gyrus (IFG/MFG), frontal pole (FP), anterior insula (AI), and inferior temporal gyrus (ITG). **(B)** Follow-up whole brain t-tests contrasting the Near versus Far revealed distinct patterns of NDE in 2nd- and 5th-graders. [**Top**] In 2nd-graders, significant distance effects were observed only in the right SMG/IPS and a frontal cluster spanning the MFG/FP. [**Bottom**] In contrast, 5th-graders showed more extensive distance effects across bilateral regions, additionally including the AI and ITG. L, Left; R, Right. The color bar represents *t*-values.

The whole-brain mixed effects ANOVA also revealed significant NDEs across three distance bins, showing greater brain activations for near distances compared to medium distances and greater activation for medium distances compared to far distances across multiple brain regions across the frontal-parietal and temporal-occipital areas (See Table S9). Notably, the largest differences were observed between the Near > Far contrasts, with extensive bilateral clusters spanning the superior parietal lobule (SPL), the intraparietal sulcus (IPS) and supramarginal gyrus (SMG), as well as the middle and inferior frontal gyrus (MFG/IFG), anterior insula (AI), frontal pole (FP), and inferior temporal gyrus (ITG) (Figure 4A). Similar regions were also identified when we restricted the analyses to the pairwise distance comparisons between near and medium distances (Near > Med), and medium and far distances (Med > Far) (See Figure S5). These NDEs remained significant even with a more stringent height threshold of *p* < 0.001. These results showed that children’s functional responses to fractions varied based on their holistic magnitudes.

To identify the brain regions sensitive to the holistic distance between fractions at each grade level, we conducted whole brain *t*-tests contrasting near and far distances (Near > Far), collapsed across all notations, for 2nd- and 5th-graders separately. Among 2nd-graders, the contrast Near > Far revealed greater activation for near distances in a parietal cluster spanning the SMG and IPS, as well as a frontal cluster including the MFG and FP (Figure 4B and Table S10). While the NDEs in 2nd-graders were localized to the right hemisphere, those in 5th-graders were more widespread, spanning the bilateral frontoparietal regions. Specifically, 5th-graders showed greater activations in bilateral clusters encompassing the SPL/IPS and SMG, along with the bilateral MFG, FP and AI, as well as the right ITG (Figure 4B and Table S10). These findings indicate that the bilateral NDEs found for the pooled sample were primarily driven by the NDEs observed in 5th-graders and further suggest distinct patterns of fraction magnitude processing between 2nd- and 5th-graders.

#### 3.2.3 | Neural Distance Effects in Each Notation Among 2nd- and 5th-Graders

**FIGURE 5:**
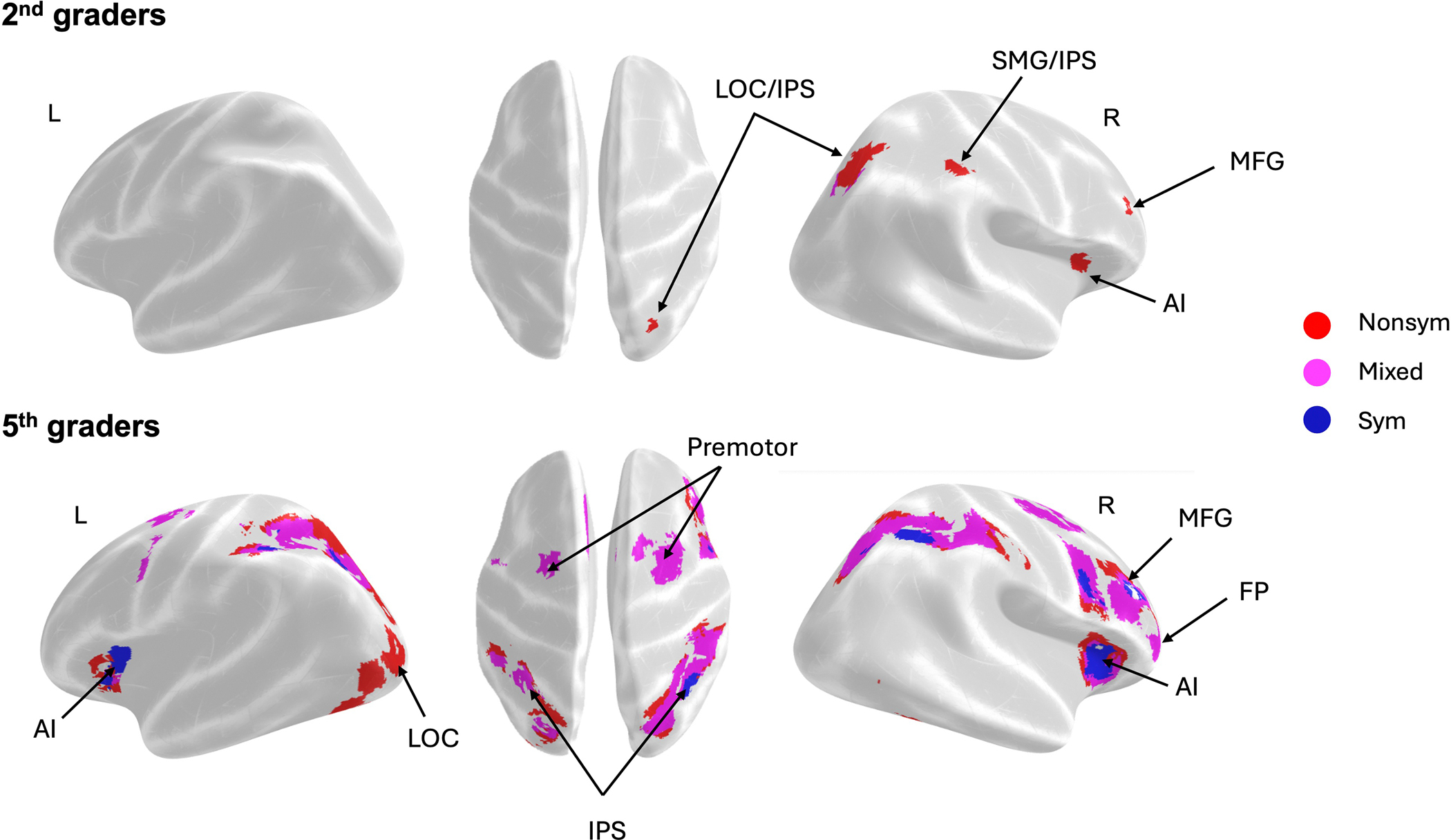
Whole-brain *t*-tests performed for each notation revealed that in 2nd-graders, significant distance effects were mainly driven by nonsymbolic (Nonsym) and mixed (Mixed) notations, but not by symbolic (Sym) notation. In contrast, 5th-graders exhibited significant neural distance effects across all three notations, with activations spanning bilateral frontal-parietal regions as observed in Figure 4 Red areas indicate regions with significant neural distance effects in response to Nonsym, magenta areas indicate regions with neural distance effects in response to Mixed, and blue areas indicate regions with neural distance effects in Sym. The brain images are inflated using the nilearn package to improve visualization of activations in the sulci (white) and gyri (gray). AI, Anterior Insula; FP, Frontal Pole, IFG, Inferior Frontal Gyrus; IPS, Intraparietal Sulcus; ITG, Inferior Temporal Gyrus; LOC, Lateral Occipital Cortex; MFG, Middle Frontal Gyrus; SMG, Supramarginal Gyrus; SPL, Superior Parietal Lobule. *Note*, the color bar represents *t*-values.

To unpack the results within each grade level, we conducted identical whole-brain analyses using the Near > Far distances within each notation. For 2nd-graders, significant NDEs were observed primarily in response to Nonsym and Mixed notations (see Figure 5 and Table S11). Significant NDEs for Nonsym were mainly in the right hemisphere, including parietal clusters spanning the lateral occipital cortex (LOC) and IPS, as well as frontal clusters encompassing the AI and MFG. Significant NDEs for Mixed were observed only in a small cluster of the LOC. Notably, the distance effects for Nonsym remained significant under a more stringent threshold of *p* < 0.001, whereas those for Mixed did not (See Supporting Information and Figure S6 for details). This series of results showed that 2nd-graders’ frontal-parietal network was more sensitive to nonsymbolic notations of fraction magnitudes than to the other notations.

In contrast to our findings with 2nd-graders, we found neural distance effects in all three notations for the 5th-grade cohort (see Figure 5; Table S12). Among 5th-graders, even Sym comparisons recruited both frontal-parietal regions including the IPS, with Nonsym and Mixed comparisons recruiting broader parietal and frontal regions. These neural distance effects remained significant under a more stringent threshold of *p* < 0.001 (See Figure S6).

Additionally, as noted in the behavioral analysis, neural distance effects might possibly result from children’s use of gap strategies and congruency levels of symbolic fraction pairs. We therefore examined whether the neural distance effects for symbolic fractions were mainly due to the holistic distance by performing a whole-brain analysis using gap rather than distance as the contrast. This analysis revealed no significant activation due to gap distance (see Supporting Information; see also Kalra et al. 2020). We also examined whether the neural distance effects for symbolic fractions were correlated with individual differences in congruency effects. While the analysis revealed significant correlations in a few small clusters, including the LOC and medial FP, these regions did not overlap with the brain areas showing significant distance effects for symbolic fraction notation (See Supporting Information and Table S13 for details). These results indicate that even before formal fraction instruction, 2nd-graders recruit fronto-parietal networks to process holistic distance between fraction magnitudes particularly when presented in nonsymbolic notation, but not when presented symbolically. In contrast, findings from 5th-graders indicate that as children receive formal instruction with fractions, they recruit parietal-frontal networks to process fraction magnitudes across all three notations.

#### 3.2.4 Developmental Differences in Neural Distance Effects Between 2nd Versus 5th Graders

**FIGURE 6:**
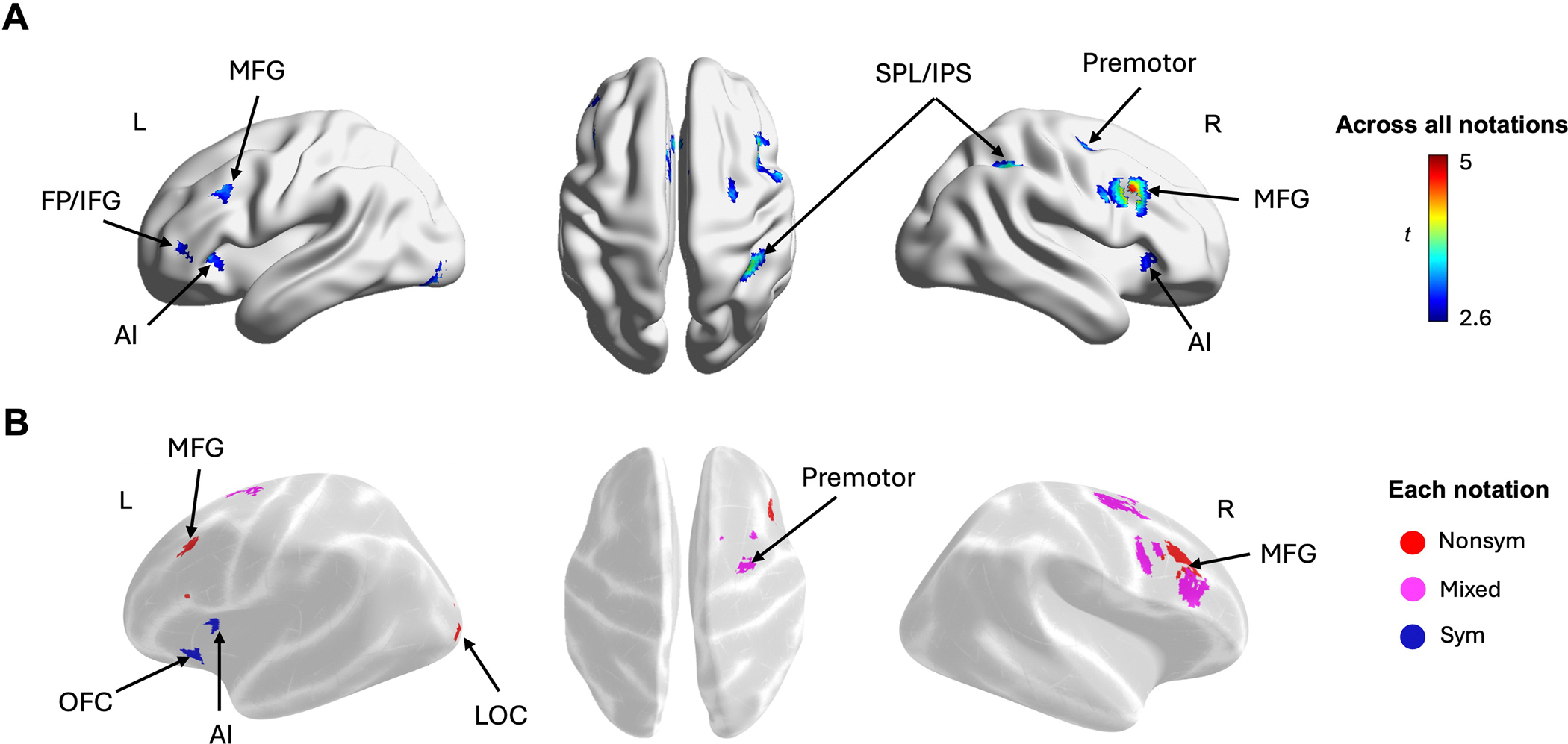
Significant interaction between Grade and Distance. **(A)** A whole-brain ANCOVA with Grade (2nd-and 5th-graders), Notation (Nonsym, Mixed, and Sym) and Distance (Near and Far) revealed greater distance effects in 5th-graders compared to 2nd-graders in multiple brain regions, including the superior parietal lobule/intraparietal sulcus (SPL/IPS), inferior and middle frontal gyrus (IFG/MFG), frontal pole (FP), and anterior insula (AI). **(B)** Follow-up analyses for each notation revealed distinctive interaction patterns. For Nonsym and Mixed notations, interactions were primarily observed in the right hemisphere, including the middle frontal gyrus (MFG), premotor cortex, and lateral occipital cortex (LOC). In contrast, interactions for Sym notation emerged in the left AI and orbitofrontal cortex (OFC). Red areas indicate regions with significant neural distance effects in response to Nonsym, magenta areas indicate regions with neural distance effects in response to Mixed, and blue areas indicate regions with neural distance effects in Sym. The brain images were inflated to improve visualization of activations in the sulci (white) and gyri (gray). The color bar represents *t*-values.

To characterize the distinctive patterns of NDEs between 2nd- and 5th-graders, we conducted a mixed-effects ANOVA to examine significant interactions between Distance (Near vs. Far) and Grade (2nd- and 5th-graders) across different notations (Nonsym, Mixed, and Sym). Specifically, 5th-graders exhibited greater NDEs compared to 2nd-graders across the frontal-parietal regions, including the right SPL/IPS and the bilateral MFG and AI (See Figure 6A; Table S14). Notably, no clusters showed greater distance effects among 2nd-graders. These interaction effects remained significant under a more stringent threshold of *p* < 0.001 (See Figure S7A). These findings suggest that older children may have developed a functional specialization for processing fraction magnitude information by engaging the fronto-parietal network more broadly compared to younger children.

To examine how these results varied by notation, we examined the interaction between NDEs and Grade within each notation condition. Significant interactions revealed that 5th-graders exhibited greater NDEs than 2nd-graders in the LOC and the MFG for Nonsym notation and in the MFG and premotor cortex for Mixed notation. While interactions for Nonsym and Mixed were primarily observed in the mid-frontal regions, those for Sym notation emerged in the left AI and orbitofrontal cortex (OFC) (See Figure 6B; Table S15). Consistent with the interactions across all notations, no clusters showed greater distance effects in 2nd-graders. Notably, the observed notation-specific effects for Nonsym and Mixed remained significant under a more stringent threshold of *p* < 0.001, whereas those for Sym did not (See Supporting Information and Figure S7B for details). These notation-specific results suggest that 5th-graders engage frontal regions more than 2nd-graders when processing both nonsymbolic and symbolic fraction magnitudes. Furthermore, the absence of interaction effects in the parietal regions for any specific notation suggests that the greater parietal engagement observed in 5th-graders compared to 2nd-graders across notations was likely driven by a combination of smaller, notation-specific interaction effects.

### 3.3 | Fraction Magnitude Representations in 2nd- and 5th-Graders: Neural Representational Similarity Analysis

Finally, to examine whether children engage similar neural patterns between nonsymbolic and symbolic fraction magnitudes, we assessed the significance of similarities (neural representational similarity, NRS) between NDEs in nonsymbolic and symbolic fractions on each set of a priori defined IPS coordinates (three right IPS and two left IPS; See Methods and Table 6). A regional-level one-sample *t*-test for each grade and IPS coordinate revealed that NRS values between nonsymbolic and symbolic NDEs significantly differed from the baseline (zero-correlation) in both 2nd- and 5th-graders, with different patterns. Among 2nd-graders, significant similarities between nonsymbolic and symbolic NDEs were primarily found in the right IPS (*t*s > 2.15, *ps* < 0.041, Cohen’s *d*s > 0.41), whereas no significant effects were found in the left IPS (|*t*s| < 0.24, *ps* > 0.816, |Cohen’s *d*s| < 0.05). In contrast, 5th-graders exhibited significant

**FIGURE 7:**
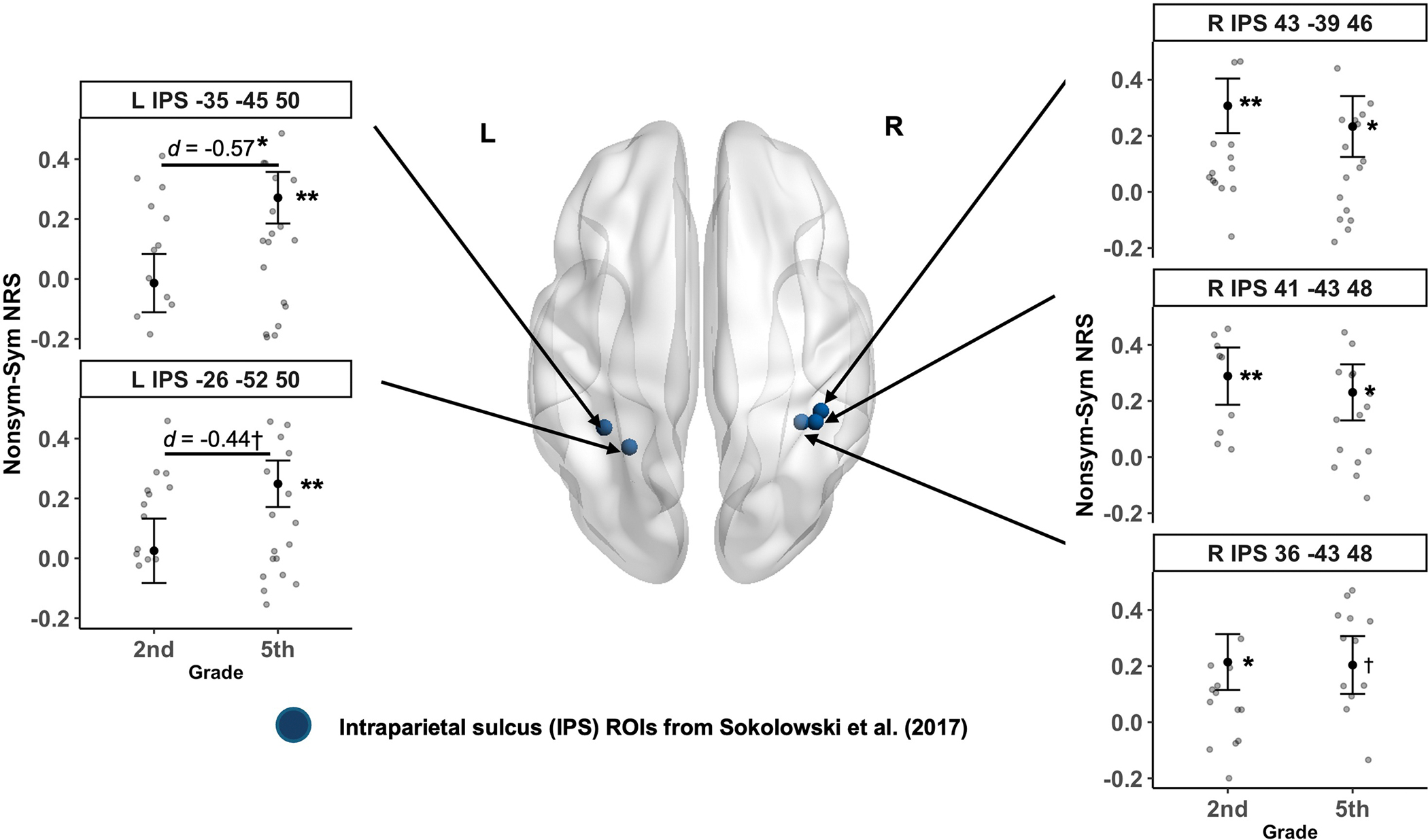
Neural representational similarity (NRS) between neural distance effects (NDEs) across nonsymbolic (Nonsym) and symbolic (Sym) notations in the intraparietal sulcus (IPS). A priori region of interest (ROI) analysis was performed using five IPS coordinates from Sokolowski et al. (2017), presented in MNI space. One-sample *t*-tests revealed significant Nonsym-Sym NRS in the left IPS coordinates for 5th-graders, but not for 2nd-graders. In contrast, both 2nd and 5th graders showed significant Nonsym-Sym NRS in the right IPS. A group *t*-tests revealed that 5th graders exhibited higher NRS values than 2nd graders in the left IPS. Asterisks* next to error bars indicate significance in the one-sample *t*-tests. +*p* < 0.10, *p < 0.05, **p < 0.01. *d* indicates Cohen’s *d*.

NRS in both left and right IPS (*t*s > 2.15, *ps* < 0.039, Cohen’s *d*s > 0.37), except for one right IPS coordinate (*t* = 1.98, *p* = 0.057, Cohen’s *d* = 0.34) (See Table 6 and Figure 7). These results indicate that 2nd-graders exhibit similar neural representations of fraction magnitudes between nonsymbolic and symbolic notations primarily in the right IPS, whereas 5th-graders represent fraction magnitudes of both notations in the bilateral IPS, similar to the observed neural distance effects in the univariate analysis.

Subsequent group *t*-tests revealed that 5th-graders exhibited higher NRS between nonsymbolic and symbolic NDEs compared to 2nd-graders, specifically in the left IPS [MNI: −26 −52 50] (*t* = −2.20, *p* = 0.032, |Cohen’s *d* | = 0.57; Table 6 and Figure 7). These findings suggest that NRS between nonsymbolic and symbolic NDEs was higher for 5th-graders, particularly in the left IPS.

## 4 Discussion

In this study, we compared behavioral performance and neural signatures for children who have not yet received formal fractions instruction (2nd-graders) and children who have received a few years of such instruction (5th-graders) to test the hypothesis that fractions knowledge builds on preexisting human capacities to process nonsymbolic fractions. Our results from both univariate and multivariate neural representational similarity analyses (RSA) were consistent with two key predictions of Lewis et al. 2016) recycling account:

1. Holistic nonsymbolic fraction processing prior to formal instruction: Children who had not yet received prior fractions instruction (i.e., 2nd-graders) could quickly and accurately compare nonsymbolic fractions and reliably recruited parietal-frontal networks—especially the right intraparietal sulcus (IPS)—to do so. Notably, 2nd-graders did not recruit these networks when comparing symbolic fractions.
2. Neuronal recycling of nonsymbolic fraction processing architectures for symbolic fraction processing: Children with prior fractions instruction (i.e., 5th-graders, in contrast to 2nd-graders) recruited similar frontal-parietal regions, especially the IPS, for both nonsymbolic fractions and symbolic fractions. Critically, 5th-graders’ multivoxel patterns of neural distance effects between nonsymbolic and symbolic fractions showed significant representational similarity.

Moreover, our univariate whole-brain and multivariate ROI data were consistent with the proposal that educational experiences may drive developmental changes in the fronto-parietal network. Below, we recap our findings, highlighting key predictions of the recycling account tested by examining similarities and differences between our cohorts. We further discuss how the patterns discovered provide new insights into the cognitive architecture for processing nonsymbolic fractional number values and how that architecture develops with the acquisition of symbolic fractions knowledge.

### 4.1 | Holistic Fraction Magnitude Processing Prior to Formal Instruction

One key tenet of the nonsymbolic fraction recycling account is that children have perceptual sensitivity to nonsymbolic analogs to fractions even prior to receiving formal instruction on symbolic numerical fractions. Our in-scanner behavioral data replicated previous findings demonstrating such sensitivity to nonsymbolic fractions among school-aged children (Kalra et al. 2020; Muñez et al. 2022; Park and Esposito 2024; Park et al. 2021), and our fMRI data allowed us, for the first time, to identify the neural systems associated with nonsymbolic fraction processing in children.

### 4.2 | Behavioral Evidence

We found that, in-scanner, 2nd-grade children could effectively compare nonsymbolic fraction magnitudes before receiving formal instruction on rational numbers. These results replicated Kalra et al. (2020)’s findings with a larger out-of-scanner cohort. Our results revealed that 2nd-graders were capable of comparing rational number magnitudes accurately and rapidly in all three notations, although 5th-graders were more accurate overall. We also found behavioral distance effects in all notations for 2nd- and 5th-graders, demonstrating that even 2nd-graders can compare holistic fraction magnitude information in multiple notations. These results were consistent with previous studies that have shown that children even as young as age 4 can process nonsymbolic fractions holistically (Park et al. 2021; Szkudlarek and Brannon 2021; see also McCrink and Wynn 2007). These findings provide strong evidence in support of the hypothesis that even very young children are equipped with a pre-existing (i.e., ontogenetically early) perceptually-based neurocognitive architecture for processing nonsymbolic fraction magnitudes.

Also as predicted, we found significant differences in performance by notation for both grade cohorts. Children were fastest and most accurate with nonsymbolic (lines vs. lines) fraction comparisons, and slowest and least accurate with symbolic (fractions vs. fractions) comparisons. The fact that nonsymbolic fraction comparisons were fast and accurate supports the idea of a pre-existing perceptually-based mechanism sensitive to nonsymbolic fraction magnitudes that may later be recycled to process symbolic fractions, as the recycling account predicts. Notably, children were just as accurate with mixed notation comparisons (lines vs. fractions) as they were with symbolic notation (fractions vs. fractions) comparisons. This is noteworthy because it was reasonable to expect that mixed notation comparisons should impose additional translation costs (Lyons et al. 2012). Specifically, if children first converted a nonsymbolic fraction to a symbolic fraction and then determined which of the two was larger, performance should presumably be worse (lower accuracy, slower reaction times) than for within format symbolic comparisons. However, this was not the case. This result replicated past findings and was consistent with our neural findings that nonsymbolic and symbolic fractions may utilize a similar representational magnitude code (See Kalra, Hubbard, and Matthews 2020; Matthews and Chesney 2015).

### 4.3 | Neuroimaging Evidence

Our univariate whole-brain analyses also provided neural evidence of holistic processing of fraction magnitudes. In children, we observed significant distance effects, with near distances eliciting the greatest activation in these frontal-parietal regions and far distances eliciting the lowest activations across all notations. Consistent with the predictions of the nonsymbolic fraction recycling account, our whole-brain analysis revealed that both 2nd-and 5th-graders showed significant neural distance effects for nonsymbolic fractions (lines-lines), with processing activating frontal-parietal regions including the IPS, the middle frontal gyrus (MFG), and the bilateral anterior insula (AI). These brain regions have previously been demonstrated to respond during nonsymbolic numerical processing, with analogs to both rational numbers (e.g., Jacob and Nieder 2009b; Mock et al. 2018) and whole numbers (e.g., Houdé et al. 2010; Sokolowski et al. 2017).

When disaggregated, these patterns diverged somewhat across grade cohorts. Second-graders exhibited right-lateralized neural distance effects, consistent with findings from other developmental studies (Ansari et al. 2005; Kersey and Cantlon 2017a). In contrast, 5th-graders showed bilateral engagement when processing nonsymbolic fraction magnitudes, similar to the patterns observed in adults (Mock et al. 2018). Altogether, these findings further buttress the argument that nonsymbolic fraction magnitudes are processed holistically in ways similar to other types of analog magnitudes by engaging similar sets of brain regions and add to our understanding of notation-dependent and independent representations (see Bulthé et al. 2014; Bulthé et al. 2015; Eger et al. 2009; Piazza and Eger 2016).

### 4.4 | Developmental Differences Between 2nd- and 5th-graders

We observed several developmental differences in response to fraction comparisons across notations. Our whole-brain ANOVA revealed multiple brain regions showing significant main effects of grade, predominantly indicating higher neural responses among 2nd-graders compared to 5th-graders during the task. Specifically, regions more heavily recruited by 2nd-graders included the right inferior frontal gyrus (IFG) and middle and superior temporal gyrus (MTG/STG), which have been implicated in cognitive control (Emerson and Cantlon 2015; Houdé et al. 2010; Kersey and Cantlon 2017b) and possibly semantics of symbolic numbers (Cohen Kadosh et al. 2005; Pinel et al. 2001). We interpret these results as suggesting that 2nd-graders require more cognitive resources—perhaps processing the semantics of fraction stimuli—to process both nonsymbolic and symbolic fractions.

On the other hand, whole-brain interactions between grade and distance indicated greater neural distance effects among 5th-graders compared to 2nd-graders. These interactions were shown in the fronto-parietal regions. Parietal areas included the right IPS and bilateral MFG and AI—regions known to be important for magnitude processing and higher-level cognitive demands (e.g., Li et al. 2022; Sokolowski et al. 2017). In addition to these magnitude-specific regions, the frontal pole likely involved processing relational information (e.g., Hartogsveld et al. 2018; Mansouri et al. 2017) and analogical reasoning (Green et al. 2009; Holyoak and Lu 2021).

Furthermore, when we examined these interactions effects by notation, we found that developmental differences varied depending on the notation type. Specifically, the greater distance effects in 5th-graders compared to 2nd-graders for nonsymbolic fractions were primarily found in the mid-frontal regions, whereas the interactions for symbolic fractions were found in the AI and orbitofrontal cortex—areas associated with domain-general cognitive control and decision-making processes (Chang et al. 2018; Kersey and Cantlon 2017a; Menon and Uddin 2010; Rudebeck and Rich 2018). These results may suggest during the early years of fraction instruction, children more strongly engage prefrontal regions to process the magnitude of symbolic fractions. Future longitudinal studies will be necessary to determine whether this prefrontal recruitment endures, or reflects a transient phase of the learning process (e.g., Rivera et al. 2005).

Taken together, these findings suggest that younger children may require broader brain engagement for fraction comparisons regardless of distances or notations, whereas older children can efficiently engage the fronto-parietal network in a distance-dependent manner. These results indicate that developmental differences in fronto-parietal networks emerge within the first few years of formal fractions instruction.

### 4.5 | Neuronal Recycling of the Nonsymbolic Fraction Processing for Symbolic Fraction Processing

The second key prediction of the nonsymbolic fraction recycling account is that symbolic fraction processing builds on preexisting neural foundations that process nonsymbolic fractions. Our univariate findings align with this prediction, revealing that 2nd-graders, who have not yet received extensive formal instruction with fractions, engaged the fronto-parietal network when processing nonsymbolic fraction magnitudes. In contrast, 5th-graders engaged the same fronto-parietal network recruited for nonsymbolic fractions—including bilateral IPS—for symbolic fraction processing. Consistent with the recycling account, symbolic fraction comparisons did not lead to significant neural distance effects among 2nd-graders, but did in 5th-graders.

Critically, the results of our regional-level neural representational similarity (NRS) analysis results accord with the recycling hypothesis by revealing significant similarity in the multivoxel patterns across nonsymbolic and symbolic distance effects in the right IPS for 2nd-graders and in the bilateral IPS for 5th-graders. These results indicate that 2nd-graders’ neural patterns for processing nonsymbolic and symbolic fraction magnitudes were similar in the right IPS—an effect not detectable in the univariate analysis. Although our findings somewhat differ from previous suggestion that the IPS encodes notation information rather than fraction magnitude per se (Bhatia et al. 2022), they have compared notation similarity with magnitude similarity across both whole numbers and fractions. Future studies should directly compare similarities in neural distance effects across nonsymbolic whole numbers and fractions to more precisely characterize fraction magnitude representations in the IPS.

Furthermore, whereas 2nd-graders did not exhibit significant NRS between nonsymbolic and symbolic fraction processing in the left IPS, 5th-graders showed significant representational similarity, with NRS values significantly higher than those observed in the 2nd-graders. These results further support the right-to-bilateral developmental shift hypothesis in symbolic fraction processing, while also reinforcing the recycling hypothesis. These findings suggest the 3 years of instruction with symbolic fractions between 2nd- and 5th-grade may facilitate the functional reorganization of the IPS—machinery initially geared to process nonsymbolic fractions—to support processing of symbolic fractions.

These developmental differences in processing fraction magnitudes are similar to those found in previous univariate studies investigating processing of numerosities. For instance, prior work has found that young children engage primarily the right IPS to process numerosities (Ansari and Dhital 2006; Cantlon et al. 2006; see also meta-analysis of Kaufmann et al. 2011). In contrast, adults engage both right and left IPS (e.g., Ansari and Dhital 2006), suggesting that the right IPS may play an important foundational role for processing numerical information early in development, with the left IPS becoming engaged later in development (Ansari 2016). Taken together, these findings demonstrate that formal fraction instruction may help engage the bilateral inferior parietal lobule, which is already specialized for nonsymbolic fractions processing, to develop neural specialization for symbolic fraction processing.

### 4.6 | Considerations Regarding the Development of Symbolic Fraction Processing

Although the patterns emerging from our data were largely consistent with the nonsymbolic fraction recycling account, they also raise a number of questions. In particular, we were struck by the fact that we did not observe neural distance effects with symbolic fractions among 2nd-graders, which stood in contrast to the fact that 2nd-graders (a) did show behavioral distance effects with symbols and (b) did demonstrate neural distance effects with mixed notation comparisons. We consider each of these issues in turn below.

### 4.7 | No 2nd-Grade Neural Responses for Symbolic Fractions Despite Behavioral Distance Effects

One plausible explanation of our failure to find neural distance effects for symbolic comparisons in the younger age group may be due to the heterogeneity of early (i.e., pre-instruction) neural responses toward symbolic fractions. Several previous studies have noted distributed neural responses in processing various information including visual stimuli as faces and even mind reading (e.g., Cox et al. 2015; Norman et al. 2006; Scherf et al. 2007). Along with these studies, computational modelling (e.g., Jacobs and Jordan 1992) and neuroimaging in non-human primates (Srihasam et al. 2012) suggest that some cognitive processes may be characterized by distributed processing early in their acquisition, which become localized to certain regions of the brain with increased experience. Based on these previous results, it is plausible that the initial representations for symbolic fractions may be sparse and broadly distributed and only converge on a dedicated neural system after formal instruction on fractions.

Thus, it is possible that in our study, individual 2nd-graders may have indeed exhibited neural distance effects for symbolic fractions, but the effects may have been too widely distributed (within individuals) and anatomically heterogeneous (across individuals) to yield localized neural distance effects in our group-level analyses. After more experience with symbolic fractions, these distributed responses may become more localized, both within and across participants, reflecting the patterns seen in the fifth graders in our study. To better understand the brain responses toward symbolic fraction in the early developing brain and developmental changes of its patterns, future studies should investigate longitudinal changes in distributed and/or overlapping representations for symbolic fraction processing.

### 4.8 | Second-Graders’ Neural Distance Effects for Mixed Comparisons But Not for Symbolic Ones

Even if the hypothesis of distributed representations for early symbolic fraction processing is correct, a question remains as to why 2nd-graders exhibited neural distance effects for mixed notations but not for within notation symbolic ones. One possible scenario is that the brain responses to nonsymbolic fractions may be more coherent and robust enough to lead to detectable neural distance effects for mixed notations. As mentioned above, neural activation for symbols can be sparse and distributed. However, sparse and distributed activation could still plausibly encode specific magnitudes, such that there was a distance dependent response for regions responsible for holistic processing of nonsymbolic fractions. This distance-dependent response may be strong enough to compensate for the weaker and distributed signals from symbolic fractions. This state of affairs would allow 2nd-graders to exhibit localized neural distance effects between holistic magnitudes in mixed notations.

Another possible scenario is that symbolic notations may allow greater trial-to-trial strategic variability than nonsymbolic notations. Children may employ a mix of strategies that are not necessarily rooted in holistic magnitude, whereas the mixed notation may force them to consistently use semantic representations that are based on holistic distance. As many previous studies have noted (e.g., Fazio et al. 2016; Morales et al. 2020; Obersteiner et al. 2013; Schneider and Siegler 2010), multiple heuristic strategies, such as choosing the stimulus with the smaller gap between numerator and denominator, often yield correct responses for symbolic fractions, but need not involve processing the holistic distance between fractions. However, these strategies cannot be used for comparing between a symbolic fraction and a nonsymbolic fraction, because there is no metric for comparing their respective gaps. Unlike symbols, comparing nonsymbolic fractions is less susceptible to use of some heuristics because of properties introduced by physical size. For example, because nonsymbolic fractions can vary in absolute physical size while maintaining the same magnitude, using the gap between components is not nearly as useful a heuristic as it is for symbolic fractions. Therefore, it is possible that mixed notation may elicit stronger neural distance effects by forcing children to more consistently use distance-dependent strategies. For more comprehensive examination of strategies used in mixed and symbolic fraction comparison condition, future studies should add additional conditions that allow examination of potential strategy such as congruency, incorporating both continuous and discretized nonsymbolic stimuli.

## 5 | Conclusion

The present study offers the first evidence demonstrating the existence of a primitive cognitive architecture for fraction processing in school-aged children at both behavioral and neural levels. This stands in contrast to theories positing that the human cognitive architecture is ill-equipped to process fractions magnitudes (e.g., Dehaene 2011; Feigenson et al. 2004), and extends the cognitive primitives account—originally proposed for whole number acquisition—to the domain of symbolic fractions. More broadly, our findings suggest that the neurocognitive architecture initially tuned only to nonsymbolic fraction magnitudes becomes tuned to symbolic fractions as children receive formal instruction about fractions. Altogether, our findings align well with the hypothesis that there is a *neuronal recycling* of nonsymbolic fraction processing architectures to acquire symbolic fraction knowledge. Future training studies would be necessary to verify this neuronal recycling account, as training studies could directly test the causal effects of different types of instruction on the neurodevelopment of symbolic fraction processing.

## Supplementary Material

Supporting Information

Additional supporting information can be found online in the Supporting Information section.

## Figures and Tables

**Table 1.** Description of Distance bins in the fraction comparison task.

Distance Bin	Mean	SD	Min	Max

Near	.144	.054	.048	.233
Medium	.341	.063	.262	.446
Far	.613	.069	.514	.750

**Table 2.** Correlations between line-lengths of components and holistic fraction values

	Numerator Incongruent	Numerator Congruent

Numerator line-lengths	.34	.83
Denominator line-lengths	−.68	−.22
Summed line-lengths	−.42	.38

* *Note*, number represents correlation coefficient (r) between the length of line components and holistic ratio values for two types of length controls

**Table 3.** Descriptive statistics for the fraction comparison task

grade	2^nd^-graders						5^th^-graders					

notation	Sym		Mixed		Nonsym		Sym		Mixed		Nonsym	

	Err	RT	Err	RT	Err	RT	Err	RT	Err	RT	Err	RT

Mean	.11	1907.82	.11	1629.33	.05	1347.02	.08	1729.73	.08	1542.82	.04	1308.85
SD	.06	304.48	.04	249.23	.03	166.29	.05	323.59	.04	289.11	.03	260.38

**Table 4.** Results from logistic mixed effects models regressing error rates for fraction comparisons against notation, reversed absolute holistic distance, and grade (i.e., 2^nd^-graders-5^th^-graders).

Regressor	*β*	*z*	*Odds Ratio*	*df*	*p*

Mixed-Nonsym	0.91***	4.92	2.48	12270	<.001
Sym-Mixed	−0.52**	−3.48	0.59	12270	.001
Absolute distance	7.53***	23.14	1860.23	12270	<.001
Grade	0.09	0.57	1.10	59	.570
Mixed-Nonsym: Absolute distance	−0.35	−0.40	0.70	12270	.690
Sym-Mixed: Absolute distance	−2.54***	−4.01	0.08	12270	<.001
Mixed-Nonsym: Grade	−0.62	−1.68	0.54	12270	.092
Sym-Mixed: Grade	0.30	0.99	1.35	12270	.323
Absolute distance: Grade	−1.42*	−2.18	0.24	12270	.029
Mixed-Nonsym: Absolute distance: Grade	−4.32*	−2.44	0.01	12270	.015
Sym-Mixed: Absolute distance: Grade	1.38	1.09	3.98	12270	.274

Note

* *p* < .05

** *p* < .01

*** *p* < .001.

**Table 5.** Results from linear mixed effects model regressing reaction times for fraction comparisons against notations, reversed absolute holistic distance, and grade (i.e., 2^nd^-graders-5^th^-graders).

Regressor	*β_std_*	*t*	*df*	*p*

Mixed-Nonsym	0.20***	10.24	11342	<.001
Sym-Mixed	0.18***	8.97	11342	<.001
Absolute distance	0.25***	32.09	11342	<.001
Grade	0.04	0.75	59	.458
Mixed-Nonsym: Absolute distance	< 0.01	−0.08	11342	.940
Sym-Mixed: Absolute distance	0.01	0.70	11342	.482
Mixed-Nonsym: Grade	0.01	0.70	11342	.485
Sym-Mixed: Grade	0.04*	2.24	11342	.025
Absolute distance: Grade	−0.05**	−2.92	11342	.004
Mixed-Nonsym: Absolute distance: Grade	−0.01	−0.39	11342	.700
Sym-Mixed: Absolute distance: Grade	0.01	0.64	11342	.524

Note

* *p* < .05

** *p* < .01

*** *p* < .001.

**Table 6.** One-sample t-tests assessing significance of neural representational similarity (NRS) between nonsymbolic and symbolic distance effects in 2^nd^- and 5^th^-graders and their differences between 2^nd^- and 5^th^-graders.

	One-sample t-test						Group t-test		
	
	2^nd^ graders			5^th^ graders			2^nd^ vs. 5^th^ graders		

ROI [MNI coordinates]	*t*	*p*	*d*	*t*	*p*	*d*	*t*	*p*	*d*

L SPL/IPS [−26 −52 50]	0.24	.816	0.05	3.21**	.003	0.56	−1.72^+^	.091	−0.44
L IPL/IPS [−35 −45 50]	−0.14	.889	−0.03	3.15**	.004	0.55	−2.20*	.032	−0.57
R IPL/IPS [41 −43 48]	2.83**	.009	0.54	2.30*	.029	0.40	0.41	.687	0.10
R IPL/IPS [43 −39 46]	3.16**	.004	0.60	2.15*	.039	0.37	0.50	.618	0.13
R IPL/IPS [36 −43 48]	2.15*	.041	0.41	1.98^+^	.057	0.34	0.07	.943	0.02

Note

+ *p* < .10

* *p* < .05

** *p* < .01.

*d* indicates Cohens’ *d*.

## Data Availability

Data that support the findings of this study are available on request from the corresponding author.

## Summary

- 2nd-graders, prior to formal fractions instructions, already recruit a right parietal-frontal network when comparing nonsymbolic fractions.
- 5th-graders, who have received some formal fractions instruction, recruit this same network not only for nonsymbolic fractions, but also for symbolic fractions.
- These findings are consistent with the neuronal recycling account, which posits that symbolic fraction processing builds on neural substrates originally used for nonsymbolic fraction processing.
- These findings suggest that pedagogical strategies focus on supporting this recycling process may enhance students’ understanding of symbolic fractions.

## Footnotes

1 Two 5th-graders (one excluded due to large movement) did not receive Power Point instruction because we did not initially provide such instructions for 5th-graders. However, we quickly learned that 5th-graders were not familiar enough with nonsymbolic ratio formats to complete the task without some guidance.
