# Supplementary material for: Developmental Changes in Nonsymbolic and Symbolic Fractions Processing: A Cross-Sectional fMRI Study
Source: Dev Sci. Author manuscript; Available in PMC 2025 Sep 1. (PMC12333650; doi:10.1111/desc.70042)
Supplement: Supporting Information [file NIHMS2097140-supplement-Supporting_Information.pdf]

Supplementary Materials

Developmental Changes in Nonsymbolic and Symbolic Fractions Processing:

A Cross-Sectional fMRI study

Supplementary Methods ………………………….…………………………... *p*. 2

Supplementary Results ………………………….…………………………. *p*. 4

Supplementary Figures……………………….………………………..........*p*. 11

Supplementary Tables.…………………..………………………………..… *p*. 19

Supplementary References.…………………..………………………………… *p*. 43

I. Supplementary Methods

1. Demographic information

The sample enrolled in the fMRI study was representative of the full behavioral sample (see details in Kalra et al., 2020). The current sample included 47 2nd-graders (21 Females) and 45 5th-graders (19 Females). Among 2nd-graders, the reported ethnicity was 2% Hispanic or Latino, 91.5% Not Hispanic or Latino, and 6.4 % Unknown or No Response. Reported race was 78.7% White, 4.3 % Asian, 10.6% Other or More Than One Race, and 6.4 % Unknown or No Response. Among 5th-graders, the reported ethnicity was 8.9 % Hispanic or Latino, 80 % Not Hispanic or Latino, 4.4% Others, and 6.7 % Unknown or No Response. Reported race was 71.1 % White, 13.3 % Other or More Than One Race, 4.4% Black or African-American, and 11.1 % Unknown or No Response. Chi-squared tests revealed no significant differences between grade groups in terms of gender, ethnicity and racial backgrounds. Additionally, our sample did not significantly differ from the full behavioral sample on these demographic variables.

2. Behavioral analysis

Previous studies suggest that participants sometimes use heuristic strategies while comparing fractions magnitudes (e.g., Fazio et al., 2016; Morales et al., 2020; Obersteiner et al., 2013; Schneider & Siegler, 2010). For example, some participants simply choose the fraction with the larger numerator or the one with the smaller denominator. Furthermore, recently, another strategy, called a '*gap*' strategy has been highlighted by a few studies (Fazio et al., 2016; Morales et al., 2020). This strategy is based on an assumption that a larger fraction has a smaller difference between its numerator and denominator (Denominator – Numerator = Gap). Take, for example, the comparison 2/9 vs. 3/5: 3/5 has a gap of 2 and 2/9 has a gap of 7, so a participant using the gap strategy would choose 3/5 due to its smaller gap. This strategy often yields a

correct answer, but there are many cases in which it does not (such as when comparing 2/3 vs. 6/8). Despite the fact that these component-based heuristics often yield incorrect responses, it is important to account for the possibility of use of componential strategies to validate the distance effect as evidence of holistic processing of fraction magnitudes. Additionally, the presence of common components in fraction comparison pairs may influence performance by allowing participants to focus on either the denominator or numerator for comparison. To address these potential confounding factors affecting distance effects, as mentioned in the main text, we conducted additional logistic and linear mixed effects using the 'gImer' and 'lmer' functions of lme4 package in R software for error rates and reaction times, respectively (Bates et al., 2014).

First, to examine the possible use of componential strategies, we performed logistic and linear mixed effects models regressing performance on symbolic comparison (error rates or reaction times) against 1) numerator, denominator, and absolute holistic distance, and against 2) 'gap' (i.e., |denominator – numerator|) distance between two fractions, absolute holistic distance, and their interaction. Notably, we did not examine interactions among numerator, denominator, and absolute distances due to collinearity issues. Consistent with the main analysis, all distances were reverse-coded by multiplying by negative one, ensuring that a smaller distance corresponded to higher error rates and reaction times.

Next, we examined the impact of congruency on distance effects for symbolic fraction comparison. We included four levels of congruency: common component congruent (CC-C), without common component congruent (WCC-C), without common component neutral (WCC-N), and without common component incongruent (WCC-IN). Similar mixed effects models were used regressing performance on each notation comparison (error rates or reaction times) against

the degree of congruency (CC-C < WCC-C < WCC-N < WCC-IN), reversed absolute holistic distance and grade, where a higher level indicated higher error rates and reaction times.

Finally, we examined the cognitive cost of notation-switching with mixed effects models, regressing performance across all notations (error rates or reaction times) taking into account switching between different notations (trials in which the notation is same as the preceding trial = 0; trials in which notation is different from the preceding trial = 1), reversed absolute holistic distance and grade.

II. Supplementary Results and Discussions

A. Behavioral Results

A1. Distance effects with binned distances

To examine behavioral distance effects, we performed mixed-effects regression analyses for both error rates and reaction times (see Methods).

Error rates. We first regressed error (incorrect: 0 or correct: 1) against grade (2 levels: 5th-graders, 2nd-graders), notation (3 levels, Nonsym < Mixed < Sym), and ordered distance bin (Far < Med < Near), such that greater regression parameters signify higher error rates (Table S2; Figure S1). Instead of using backward differencing coding, we have treated the binned distance condition as a linearly ordered factor to ensure consistency with the absolute distance results. As predicted, we found a significant effect of grade, whereby 2nd-graders were more likely to make errors than 5th-graders (*Odds Ratio* (OR) = 1.69, p = .001). Critically, we identified significant distance effects: the likelihood of making an error was progressively decreasing across the Far, Med, and Near distance conditions (OR = 8.19, $p < 0.001$). Across notations, the likelihood of making an error for Mixed was higher than for Nonsym (OR = 2.24, $p < .001$), and the likelihood

of making an error for Sym was higher than that for Mixed (*OR* = 1.46, *p* = .008). Additionally, we found significant interactions in error rate between grade and Nonsym-Mixed differences, suggesting that 5th-graders performed similarly across Nonsym and Mixed, whereas 2nd-graders showed higher error rates for Mixed compared to Nonsym with greater differences.

Reaction times. Contrary to expectations, analysis with reaction times showed that 5th-graders were not significantly faster than 2nd-graders ($\beta_{std} = 0.08$, $p = .102$) (Table S3; Figure S1). However, other effects mirrored error rate analyses. We found a significant distance effect for reaction times, with participants responding progressively slower across the Far, Med, and Near distance condition ($\beta_{std} = 0.24$, $p < .001$). Furthermore, both grade cohorts responded faster for Nonsym than for Mixed notations ($\beta_{std} = 0.20$, $p < .001$), and responded faster for Mixed than for Sym notation ($\beta_{std} = 0.17$, $p < .001$). We also confirmed significant interactions between grade and notation differences (Mixed - Nonsym: $\beta_{std} = 0.02$, *ps* < .029; Sym - Mixed: $\beta_{std} = 0.03$, *ps* < 0.001), and between grade and distance ($\beta_{std} = - 0.02$, *ps* < .001).

A2. The effects of componential strategies in symbolic fraction comparisons

To evaluate the effects of potential heuristic strategies during symbolic fraction comparisons, we performed mixed effects regression analyses regressing error rates and reaction times against each potential confounding factor, absolute holistic distance, and grade.

Numerator or denominator distances We observed significant distance effects in children for both error rates (*OR* = 998.97, $p < .001$) and reaction times ($\beta_{std} = 0.21$, $p < .001$) even after controlling for numerator and denominator distances (See Table S4). The effect of numerator and denominator distances were also significant (*ps* < .001), especially in error rates. Note that the directionality was different from the distance effects (*ORs* < 1.00), such that smaller numerator

and denominator distances predicted lower likelihood of making error rates, likely due to higher negative correlations in fixed effects between holistic distance and numerator distance (r = -0.70).

Gap distance We observed significant distance effects in both 2nd- and 5th-graders even after controlling for the gap distances in both measures (See Figure S2, Table S5). Our data showed that absolute holistic distance between compared fractions significantly explained children's both error rates (OR = 2044.24, $p < .001$) and reaction times (β_{std} = 0.30, $p < .001$) even when controlling for gap distances. Gap distance significantly explained only children's error rates (OR = 1.14, p = .045), not reaction times (p = .773), with much smaller likelihood compared to absolute distance. Also, the interaction between gap distance and the distance effect was significant in error rates (OR = 1.72, p = .014), but not in reaction times (p = .319). These results led us to examine potential effects of gap strategy in the neural distance effects within fraction comparisons (see Results section B.1).

Taken together, these results confirmed the significance of distance effects even when accounting for the effects of various componential strategies. The significant effects of holistic distances showed that both grade children did utilize holistic distances between two magnitudes to choose a larger fraction.

A3. The effects of cognitive demands

Congruency effect To evaluate the potential impact of congruency on each nonsymbolic and symbolic notation comparison, we performed logistic and mixed effects regression examining fixed effects by regressing error rates and reaction times against congruency, absolute holistic distance, and grade. All regression models for error rates and reaction times confirmed

significant distance effects (error rates: OR =112.38, $p < .001$; reaction times: $\beta_{std} = 0.30$, $p < .001$), even after controlling for congruency, grade, and all the interactions among regressors. Additionally, we found a significant effect of congruency in both error rates ($OR = 4.16$, $p < .001$) and reaction times ($\beta_{std} = 0.10$; $p = .003$), as well as interaction between absolute holistic distance and congruency in reaction times ($\beta_{std} = 0.15$; $p = .004$) (See Table S6; Figure S3). These results led us to examine the potential effects of congruency on neural distance effects within fraction comparisons (see Results section B.2).

The effects of notation switching demands across trials Lastly, to evaluate possible effects of cognitive demands related to switching of notations, we performed logistic and linear mixed model regressions investigating how trial type (switch vs. non-switch) affected error rates and reaction times compared to the effects of distance and grade. For error rates and reaction times, we found that distance effects remained significant (error rates: $OR = 936.66$, $p < .001$; reaction times: $\beta_{std} = 0.26$, $p < .001$), even after controlling for notation switching, grade, and all the interactions among regressors (Table S7). Critically, we found no significant effects for task switching ($ps > 0.645$), nor did we find a task switch-by distance interaction ($ps > .078$) for either error rates or for reaction times.

In conclusion, these results confirmed that the observed distance effects were stable even after accounting for the potential impacts of congruency effects and notation switching demands.

B. Neuroimaging Results

B1. The neural effects of gap strategy in symbolic fraction comparisons

To address the possibility that gap differences were the driving force behind our observed neural response to the distances between fraction magnitudes, we performed a first-level analysis similar to that described in the main text using gap rather than distance as the contrast for symbolic comparisons trials. Gaps were binned into "Small" (0-1), "Medium" (2-3), and "Large" (4-6) bins. Brain responses were modeled based on correct trials for each gap rather than distance (gap distances: Small, Medium, Large; notations: Nonsym, Mixed, and Sym), using a boxcar function matching the trial duration and convolved with a canonical hemodynamic response function. After generating voxel-wise contrast maps for each participant using the GLM, we conducted whole-brain group *t*-tests to examine the Small > Large contrast. The one-sample t-test revealed no significant voxels showing greater activation in the Small gap distance bin compared to the Large gap distance. These results suggest that the observed neural distance effects were not driven by the gap distances between the two fractions.

B2. The neural effects of congruency effect in symbolic fraction comparisons

The behavioral analysis showed that the distance effects measured by reaction times during fraction comparison could be partly explained by congruency. To evaluate whether the congruency of fraction stimuli contributed to the observed neural distance effects, we performed a whole-brain analysis across both grade levels, testing a correlation between neural distance effects for symbolic fractions and individual differences in congruency (indexed by differences in reaction times between congruent and incongruent conditions; CC-C + WCC-C vs. WCC-IN), included as a covariate. Our brain-behavioral correlation analysis revealed a significant positive correlation between neural distance effects and congruency effects in small clusters within the

left lateral occipital cortex (LOC), paracingulate gyrus (PaCG), and medial frontal pole (FP) (See Table S11). Specifically, the medial FP has been associated with monitoring and redistributing cognitive control resources (Mansouri et al., 2017), whereas the lateral FP—the area showing significant distance effects (see Figure 4)—is reported to be involved in processing relational information (Hartogsveld et al., 2018) and analogical reasoning (Green et al., 2009; Holyoak & Lu, 2021). Most importantly, none of these regions overlapped with the frontal-parietal network exhibiting the neural distance effects. These results suggest that the neural distance effects in fraction comparisons observed in our univariate analysis for 5th-graders were less likely to be influenced by congruency effects which are thought to engage cognitive control.

B3. Significant results at a more stringent threshold of uncorrected *p* < .001.

We would like to highlight a few results that did not persist when we used a more stringent threshold of uncorrected $p < 0.001$ ($k = 30$).

Main effect of grade The whole-brain mixed-design ANOVA was conducted with Grade (2nd- and 5th-graders) as a between-subjects factor and Notation (Nonsym, Mixed, Sym) and Distance (Near, Medium, Far) as within-subjects factors, using a threshold of uncorrected $p < .001$ (k = 30). We confirmed that the greater activation observed in 2nd-graders compared to 5th-graders, as reported in the main text, remained significant even under a more stringent height threshold of uncorrected $p < .001$ (Figure S4). However, the regions showing greater activation in 5th-graders compared to 2nd-graders were no longer significant.

Main effect of distance We confirmed that the main effect of distance remained significant with a threshold of uncorrected $p < .001$ ($k = 30$). Specifically, a whole-brain t-test contrasting Near vs. Far within each notation, using a more stringent initial threshold, revealed that significant

distance effects persisted for Nonsym in 2nd-graders. However, these effects were no longer present for Mixed, diverging from the findings based on the initial uncorrected thresholded of $p < .005$. The absence of neural distance effects for Sym was consistent with the results reported in the main text. On the other hand, significant neural distance effects were consistently observed in 5th-graders across all notations (see Figure S6).

Interaction between grade and distance We confirmed that the interaction effect between grade (2nd- and 5th-graders) and distance (Near vs. Far) remained significant in the frontal-parietal regions with a threshold of uncorrected $p < .001$ (See Figure S6). Regarding notation-specific interactions, interactions within Nonsym and Mixed notation remained significant but were confined to restricted regions, whereas interactions within Sym were no longer significant (See Figure S7).

III. Supplementary Figures

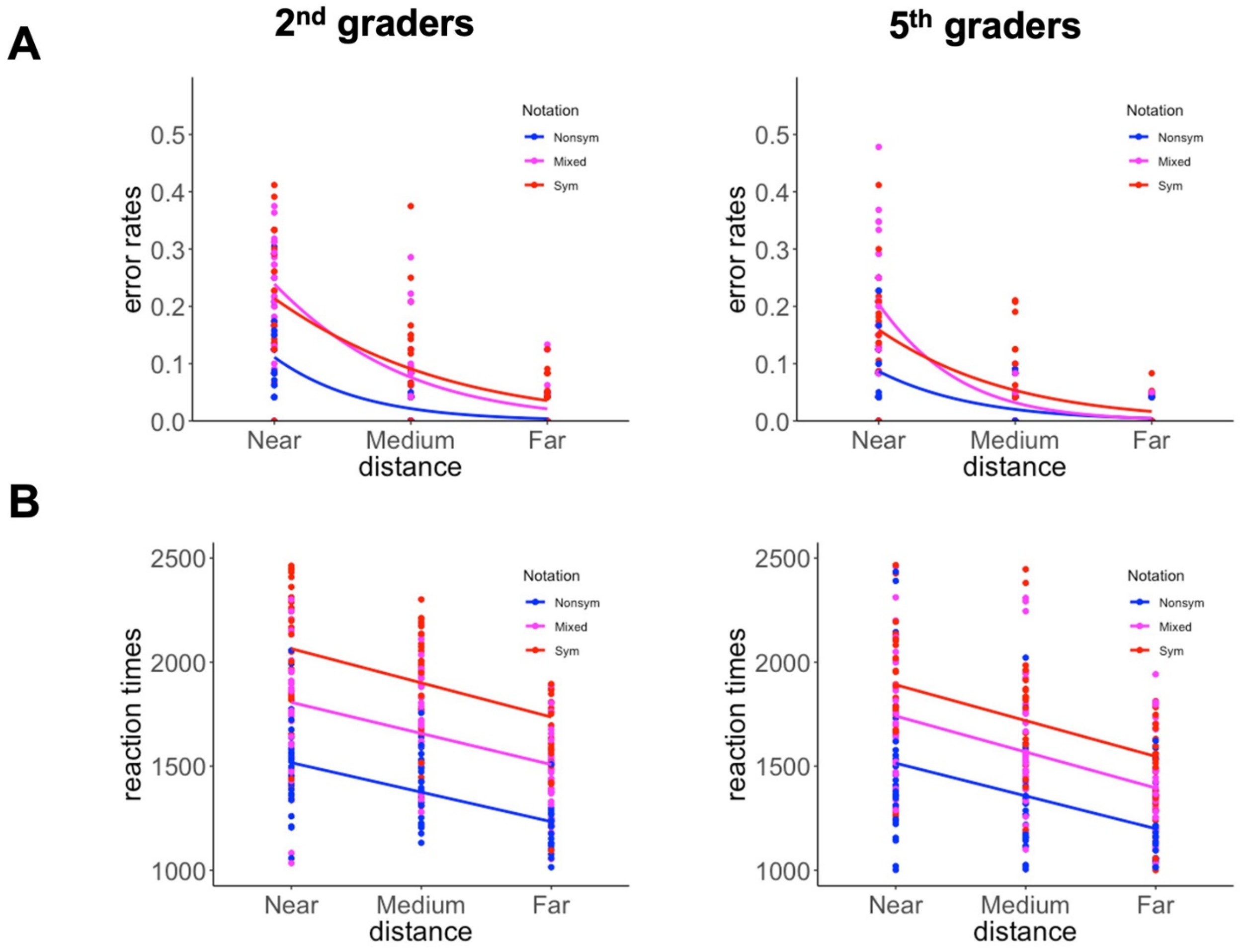

Figure S1. Displays of (A) error rates and (B) reaction times of 2nd-graders' (left) and 5th-graders' (right) performance by notation (Nonsym, Mixed, and Sym) and distance bin (Near, Medium, and Far). Each point indicates an individual's mean performance. Both grades exhibited significant distance effects such that error rates and reaction times increased as the distance between two fraction magnitude pairs decreased (*ps* < .001), and significant notation effects (*ps* < .001) whereby error rates and reaction times increased in the order of nonsymbolic (Nonsym), mixed (Mixed) and symbolic (Sym) notations of ratio magnitude comparisons.

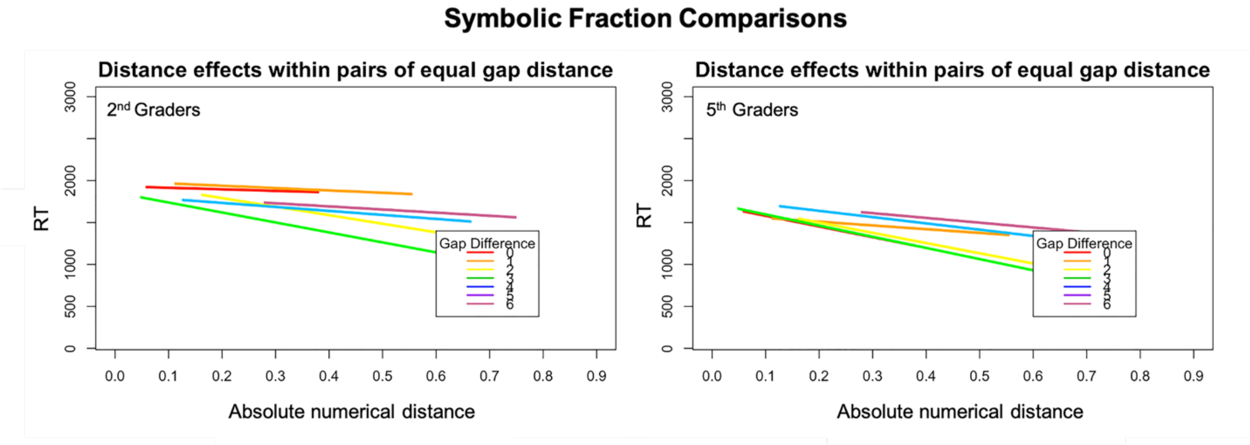

Figure S2. Distance effect slopes across gap differences for symbolic fractions in 2nd- (left panel) and 5th-graders. Significant distance differences were found in both graders even when controlling for gap distances (*ps* < .001)

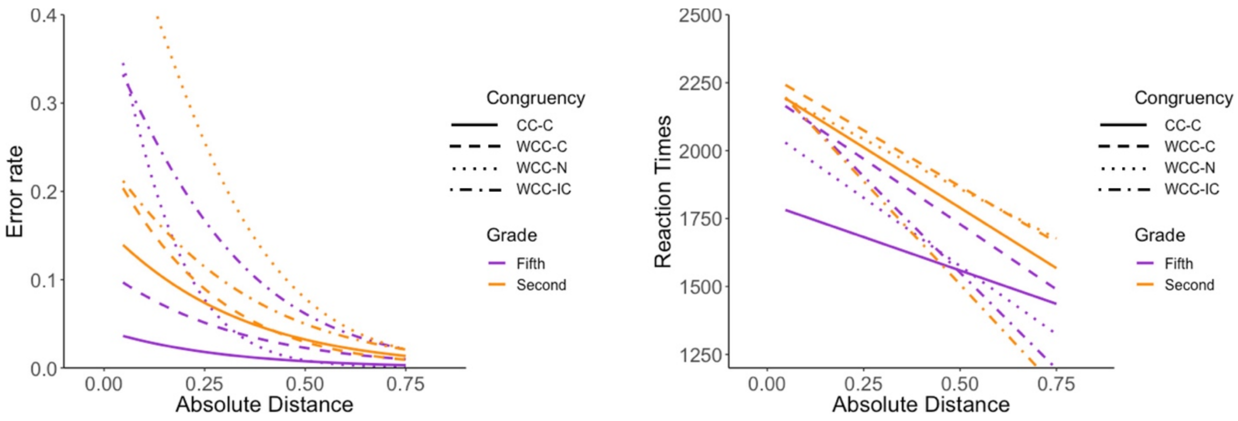

Figure S3. Distance effects for symbolic fractions across the levels of congruency of the fraction stimuli and grades in error rates (left panel) and reaction times (right panel). Abbreviations: CC-C, Common Component Congruent; WCC-C, Without Common Component Congruent; WCC-N, Without Common Component Neutral; WCC-IN, Without Common Component Incongruent.

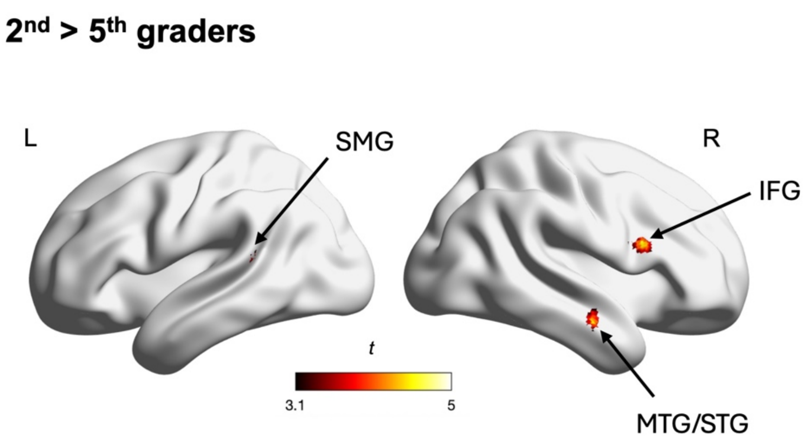

Figure S4. Significant main effect of grade identified by whole brain mixed-design ANOVA with Grade (2nd- and 5th-graders), Notation (Nonsym, Mixed, Sym) and Distance (Near, Medium, Far) using a more stringent height threshold of $p < .001$ ($k = 30$), with multiple comparisons corrected using the FWE threshold of $p < .05$. Consistent with the results reported in the main text, the greater activation in 2nd-graders compared to 5th-graders remained significant even when applying more stringent height threshold. While the cluster sizes were slightly reduced, significant differences were still observed in the left supramarginal gyrus (SMG), a cluster spanning the right middle and superior temporal gyrus (MTG and STG), and the right inferior frontal gyrus (IFG). In contrast, the regions showing greater activation in 5th-graders compared to 2nd-graders were no longer significant under stricter threshold. *Note*, the color bar represents *t*-values.

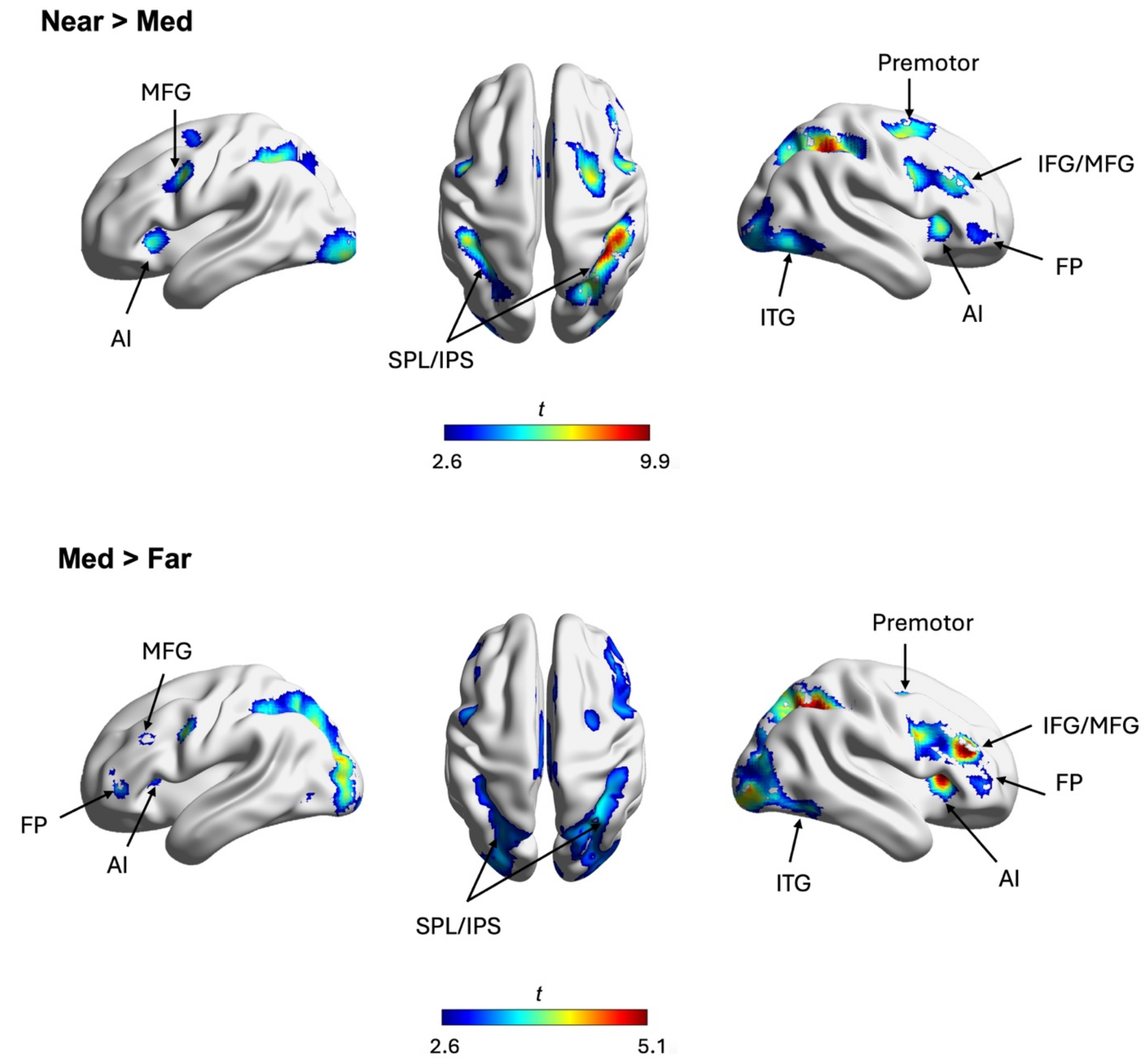

Figure S5. Significant distance effects between near and medium distances and between medium and far distances, controlling for notation (Nonsym, Mixed, Sym) and grade. **(A)** A whole brain mixed-design ANOVA revealed significant effects in the Near vs. Med contrasts across multiple brain regions similar to the Near vs. Far contrast (Figure 4A). These regions largely overlapped with those identified in the Near vs. Far contrast and included the bilateral superior parietal lobule (SPL), inferior parietal sulcus (IPS), inferior and middle frontal gyrus (IFG/MFG), frontal pole (FP), and anterior insula (AI), as well as the right inferior frontal gyrus (IFG) and inferior

temporal gyrus (ITG). **(B)** The same ANOVA model identified significant effects in the Med vs. Far contrasts, largely in similar brain regions similar to those observed in the Near vs. Med and Near vs. Far contrasts. Abbreviations: L, Left; R, Right. *Note*, the color bar represents *t*-values.

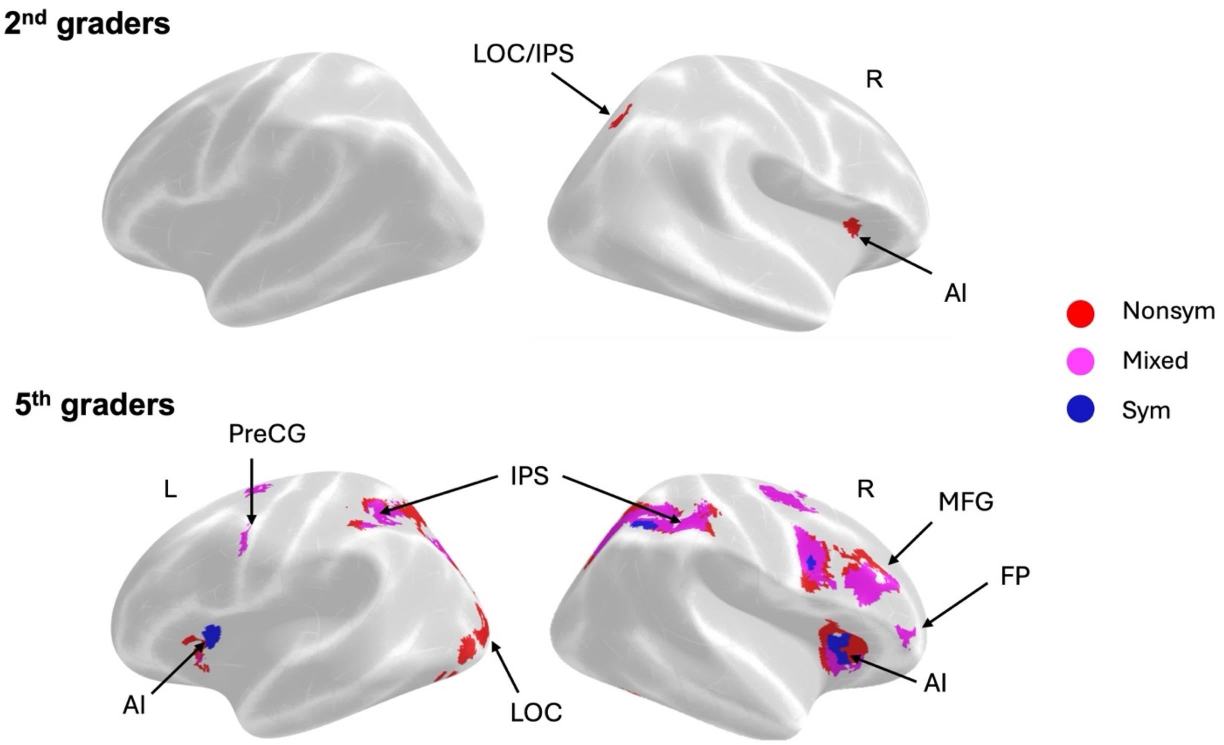

Figure S6. Significant neural distance effect by notation in 2nd-graders [top] and 5th-graders [bottom] using a more stringent height threshold of $p < .001$ ($k = 30$), with multiple comparisons corrected using the FWE threshold of $p < .05$. A whole-brain t-test with a more stringent initial threshold, contrasting binned distance (Near vs. Far) in each notation, revealed significant distance effects for nonsymbolic (Nonsym) comparisons in 2nd-graders, but not for mixed notations of nonsymbolic and symbolic (Mixed) and symbolic (Sym) comparisons. On the other hand, significant neural distance effects were consistently observed in 5th-graders across all notations. Red areas indicate regions with neural distance effects in response to Nonsym, magenta areas indicate regions with neural distance effects in response to Mixed, and blue areas indicate regions with neural distance effects in Sym. The brains are inflated to allow visualization of activations in the sulci (white) and gyri (gray). Abbreviations: AI, Anterior Insula; FP, Frontal Pole, IFG, Inferior Frontal Gyrus; IPS, Intraparietal Sulcus; LOC, Lateral

Occipital Cortex; MFG, Middle Frontal Gyrus; PreCG, Precentral Gyrus; L, Left; R, Right. *Note*, the color bar represents *t*-values.

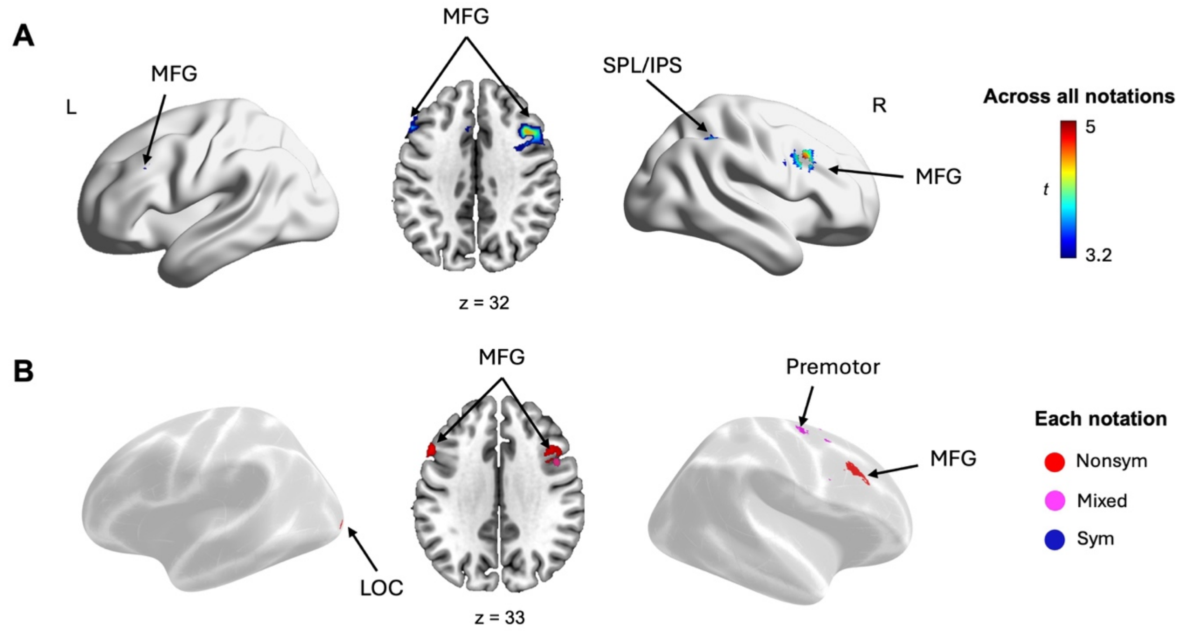

Figure S7. Significant interaction between Grade and Distance using a more stringent threshold of $p < .001$ ($k = 30$), with multiple comparisons corrected using the FWE threshold of $p < .05$. **(A)** A whole-brain ANOVA with Grade (2nd-and 5th-graders), Notation (Nonsym, Mixed, and Sym) and Distance (Near and Far) revealed greater distance effects in 5th-graders compared to 2nd-graders remained significant in the superior parietal lobule/intraparietal sulcus (SPL/IPS) and middle frontal gyrus (MFG). **(B)** The ANOVA performed within each notation using a more stringent threshold revealed that interactions within Nonsym and Mixed notation remained significant but were confined to restricted regions, whereas interactions within Sym were no longer significant. Red areas indicate regions with significant neural distance effects in response to Nonsym, magenta areas indicate regions with neural distance effects in response to Mixed, and blue areas indicate regions with neural distance effects in Sym. The brain images are inflated to improve visualization of activations in the sulci (white) and gyri (gray). *Note*, the color bar represents *t*-values.

IV. Supplementary Tables

Table S1. Symbolic Fraction Stimuli

Item	F1	F2	F1: Decimal Value	F1 Gap (Den- Num)	F2: Decima l Value	F2 Gap (Den - Num)	Gap distance (F1 Gap - F2 Gap)	Absolute Distance	Binned Distance	Common Components	Component Congruency
7/9_8/9	7/9	8/9	0.78	2	0.89	1	1	0.11	Near	CC	congruent
3/7_4/7	3/7	4/7	0.43	4	0.57	3	1	0.14	Near	CC	congruent
2/5_3/5	2/5	3/5	0.40	3	0.60	2	1	0.20	Near	CC	congruent
3/7_5/7	3/7	5/7	0.43	4	0.71	2	2	0.29	Med	CC	congruent
2/5_4/5	2/5	4/5	0.40	3	0.80	1	2	0.40	Med	CC	congruent
1/9_5/9	1/9	5/9	0.11	8	0.56	4	4	0.45	Med	CC	congruent
2/7_6/7	2/7	6/7	0.29	5	0.86	1	4	0.57	Far	CC	congruent
1/5_4/5	1/5	4/5	0.20	4	0.80	1	3	0.60	Far	CC	congruent
1/6_5/6	1/6	5/6	0.17	5	0.83	1	4	0.67	Far	CC	congruent
1/8_7/8	1/8	7/8	0.13	7	0.88	1	6	0.75	Far	CC	congruent
4/5_6/7	4/5	6/7	0.80	1	0.86	1	0	0.06	Near	WCC	congruent
3/5_5/7	3/5	5/7	0.60	2	0.71	2	0	0.11	Near	WCC	congruent
2/3_5/6	2/3	5/6	0.67	1	0.83	1	0	0.17	Near	WCC	congruent
1/7_4/9	1/7	4/9	0.14	6	0.44	5	1	0.30	Med	WCC	congruent
1/4_5/8	1/4	5/8	0.25	3	0.63	3	0	0.38	Med	WCC	congruent
1/3_5/7	1/3	5/7	0.33	2	0.71	2	0	0.38	Med	WCC	congruent
1/4_7/9	1/4	7/9	0.25	3	0.78	2	1	0.53	Far	WCC	congruent
1/3_8/9	1/3	8/9	0.33	2	0.89	1	1	0.56	Far	WCC	congruent
1/4_8/9	1/4	8/9	0.25	3	0.89	1	2	0.64	Far	WCC	congruent
1/7_7/8	1/7	7/8	0.14	6	0.88	1	5	0.73	Far	WCC	congruent
1/8_2/7	1/8	2/7	0.13	7	0.29	5	2	0.16	Near	WCC	neutral
4/9_5/8	4/9	5/8	0.44	5	0.63	3	2	0.18	Near	WCC	neutral
1/6_2/5	1/6	2/5	0.17	5	0.40	3	2	0.23	Near	WCC	neutral
5/9_7/8	5/9	7/8	0.56	4	0.88	1	3	0.32	Med	WCC	neutral
2/9_3/5	2/9	3/5	0.22	7	0.60	2	5	0.38	Med	WCC	neutral
1/8_4/7	1/8	4/7	0.13	7	0.57	3	4	0.45	Med	WCC	neutral
1/9_5/8	1/9	5/8	0.11	8	0.63	3	5	0.51	Far	WCC	neutral
1/5_3/4	1/5	3/4	0.20	4	0.75	1	3	0.55	Far	WCC	neutral
2/9_6/7	2/9	6/7	0.22	7	0.86	1	6	0.64	Far	WCC	neutral
1/7_5/6	1/7	5/6	0.14	6	0.83	1	5	0.69	Far	WCC	neutral

2/7_1/3	2/7	1/3	0.29	5	0.33	2	3	0.05	Near	WCC	incongruent
3/8_1/2	3/8	1/2	0.38	5	0.50	1	4	0.13	Near	WCC	incongruent
5/9_3/4	5/9	3/4	0.56	4	0.75	1	3	0.19	Near	WCC	incongruent
2/9_1/2	2/9	1/2	0.22	7	0.50	1	6	0.28	Med	WCC	incongruent
3/8_2/3	3/8	2/3	0.38	5	0.67	1	4	0.29	Med	WCC	incongruent
4/9_3/4	4/9	3/4	0.44	5	0.75	1	4	0.31	Med	WCC	incongruent

*Adapted from Kalra et al. (2020), Abbreviation: CC, Common Component; F, Fraction; Den, Denominator; Num, Numerator; WCC, Without Common Component.

Table S2. Results from logistic mixed effects models regressing error rates for fraction comparisons against notation (i.e., Nonsym, Mixed, and Sym), binned distance (i.e., Far < Med < Near), and grade (i.e., 2nd-graders - 5th-graders).

	β	*z*	*Odds Ratio*	*df*	*p*
Mixed-Nonsym	**0.81**	4.35	2.24	12264	<0.001
Sym-Mixed	**0.38**	2.65	1.46	12264	0.008
Distance	**2.10**	16.41	8.19	12264	<0.001
Grade	**0.53**	3.33	1.69	59	0.001
Mixed-Nonsym: Distance	0.38	1.06	1.46	12264	0.289
Sym-Mixed: Distance	**-0.8**	-2.86	0.45	12264	0.004
Mixed-Nonsym: Grade	**0.82**	2.23	2.28	12264	0.026
Sym-Mixed: Grade	-0.27	-0.96	0.76	12264	0.335
Distance:Grade	-0.28	-1.09	0.76	12264	0.275
Mixed-Nonsym: Distance: Grade	-1.34	-1.9	0.26	12264	0.058
Sym-Mixed: Distance: Grade	0.64	1.14	1.89	12264	0.252

Table S3. Results from linear mixed effects models regressing reaction times for fraction comparisons against notation (i.e., Nonsym, Mixed, and Sym), binned distance (i.e., Far < Med < Near), and grade (i.e., 2nd-graders - 5th-graders).

	β_{std}	*t*	*df*	*p*
Mixed-Nonsym	**0.20**	22.12	11336	<0.001
Sym-Mixed	**0.17**	18.14	11336	<0.001
Distance	**0.24**	30.45	11336	<0.001
Grade	0.08	1.61	59	0.107
Mixed-Nonsym: Distance	0.00	0.26	11336	0.791
Sym-Mixed: Distance	0.00	0.20	11336	0.842
Mixed-Nonsym: Grade	**0.02**	2.18	11336	0.029
Sym-Mixed: Grade	**0.03**	3.77	11336	<0.001
Distance:Grade	**-0.02**	-2.69	11336	0.007
Mixed-Nonsym: Distance: Grade	0.00	-0.49	11336	0.623
Sym-Mixed: Distance: Grade	0.01	0.76	11336	0.449

Table S4. Logistic and linear mixed effects that regressed error rates and reaction times, respectively, for symbolic fraction comparisons against absolute, numerator, and denominator distances, and grade (i.e., 2nd-graders - 5th-graders).

	Error Rates					Reaction times			
Regressor	β	z	*Odds Ratio*	df	p	β_{std}	t	df	p
Absolute distance	**6.91**	13.02	998.97	4011	<0.001	**0.21**	9.05	3525	<0.001
Numerator distance	**-0.21**	3.7	0.81	4011	<0.001	**-0.05**	-2.00	3525	0.045
Denominator distance	**-0.15**	5.66	0.86	4011	<0.001	0.02	1.41	3525	0.160
Grade	**0.53**	3.05	1.69	59	0.002	**0.14**	2.24	59	0.029

Note, significant results are bolded.

Table S5. Logistic and linear mixed effects that regressed error rates and reaction times for symbolic fraction comparisons, respectively, against absolute distance, gap distance and grade (i.e., 2nd-graders - 5th-graders).

	Error Rates					Reaction times			
Regressor	*β*	*z*	*OR*	*df*	*p*	β_{std}	*t*	*df*	*p*
Absolute distance	**7.62**	8.97	2044.24	4008	<0.001	**0.30**	9.97	3630	<0.001
Gap distance	**0.13**	2.00	1.14	4008	0.045	0.01	0.29	3630	0.773
Grade	0.18	0.51	1.20	59	0.611	**0.18**	2.26	59	0.024
Absolute distance: Gap distance	**0.54**	2.45	1.72	4008	0.014	-0.05	-1.00	3630	0.319
Absolute distance: Grade	**-6.82**	-4.02	< 0.01	4008	<0.001	-0.07	-1.05	3630	0.294
Gap distance: Grade	**0.35**	2.74	1.42	4008	0.006	**-0.18**	-2.87	3630	0.004
Absolute distance: Gap distance: Grade	-0.74	-1.67	0.48	4008	0.094	-0.06	-0.89	3630	0.375

Note, significant results are bolded. Abbreviations: OR, Odds Ratio.

Table S6. Logistic and linear mixed effects that regressed error rates and reaction times, respectively, against absolute distance, congruency levels (congruent < neutral < incongruent), and grade (i.e., 2nd-graders - 5th-graders).

	Error Rates					Reaction times			
	β	*z*	*OR*	*df*	*p*	β_{std}	*t*	*df*	*p*
Absolute distance	**4.72**	10.00	112.38	4008	<0.001	**0.30**	13.42	3627	<0.001
Congruency	**1.42**	4.90	4.16	4008	<0.001	**0.10**	2.97	3628	0.003
Grade	0.57	1.86	1.76	59	0.063	0.12	1.82	107	0.069
Absolute distance: Congruency	1.09	1.06	2.98	4008	0.290	**0.15**	2.9	3627	0.004
Absolute distance: Grade	-0.76	-0.81	0.47	4008	0.420	0.01	0.16	3627	0.873
Congruency: Grade	**-1.51**	-2.59	0.22	4008	0.009	**-0.10**	-2.76	3628	0.006
Absolute distance: Congruency: Grade	-1.65	-0.80	0.19	4008	0.423	-0.03	-0.64	3627	0.522

Note, significant results are bolded. Abbreviations: OR, Odds Ratio

Table S7. Logistic and linear mixed effects that regressed reaction times for fraction comparisons against absolute distance, notation switching, and grade (i.e., 2nd-graders - 5th-graders).

	Error Rates					Reaction times			
	β	z	*OR*	*df*	p	β_{std}	t	*df*	p
Absolute distance	**6.84**	23.63	936.66	11930	<0.001	**0.24**	26.74	11020	<0.001
Notation Switching	-0.06	-0.43	0.94	11930	0.665	-0.01	-0.46	11020	0.645
Grade	0.06	0.4	1.07	59	0.692	0.03	0.55	11020	0.581
Absolute distance: Notation Switching	-1.02	-1.76	0.36	11930	0.078	-0.02	-0.89	59	0.373
Absolute distance: Grade	**-1.82**	-3.15	0.16	11930	0.002	**-0.05**	-2.77	11020	0.006
Notation Switching: Grade	0.4	1.44	1.50	11930	0.150	<0.01	-0.13	11020	0.894
Grade: Notation Switch: Grade	1.93	1.68	6.90	11930	0.094	<0.01	-0.02	11020	0.982

Note, significant results are bolded. Abbreviations: OR, Odds Ratio

Table S8. Brain regions showing significant main effects of the group (2nd- vs. 5th-graders).

Region	Cluster Size	T-value	MNI coordinates x	y	z
5th > 2nd graders					
L COper	85	3.67	-44	-2	10
L Heschl's gyrus		3.10	-46	-12	8
L STG		2.70	-42	-20	0
2nd > 5th graders					
R IFG	254	4.77	48	14	18
R Precuneus	470	4.77	10	-58	42
R CG		3.71	0	-46	38
R CG		2.99	18	-46	36
R MTG	219	4.69	62	-10	-18
R MTG		3.10	52	-8	-24
R STG		2.93	44	-12	-12
R LOC	131	3.74	28	-72	58
R Precuneus		3.39	6	-70	56
L OFG	347	4.81	-12	-78	-18
R LG		4.03	6	-80	-14
L SMG	114	4.39	-58	-44	12

Abbreviations: CG, Cingulate Gyrus; COper, Central Operculum Cortex; IFG, Inferior Frontal Gyrus; LG, Lingual Gyrus; LOC, Lateral Occipital Cortex; MTG, Middle Temporal Gyrus;

OFG, Orbitofrontal Cortex; SMG, Supramarginal Gyrus; STG, Superior Temporal Gyrus; L, Left; R, Right.

Table S9. Brain regions showing significant main effect of distance.

Region	Cluster Size	T-value	MNI coordinates x	y	z
Near > Far					
R SPL/IPS	16887	14.11	38	-48	46
R SMG/IPS		13.01	44	-40	50
R LOC		11.73	30	-64	36
R Premotor/PaCG	13327	11.18	0	18	48
R AI		11.07	32	22	2
R PreCG		10.8	56	10	38
L PreCG	1425	10.24	-46	2	36
L MFG		6.15	-48	30	36
R MFG		4.18	-44	26	28
R SFG	217	4.89	-26	-2	60
R Brain Stem	86	4.73	2	-24	-10
R Thalamus		3.27	8	-20	-4
L HIPP	141	3.71	-26	-34	6
L Thalamus/HIPP		3.55	-20	-30	-4
L HIPP		2.86	-32	-34	0
R PreCG	80	3.62	4	-32	72
Near > Med					
R SPL/IPS	6130	9.85	36	-46	44
R SMG/IPS		9.42	44	-40	50
R LOC		7.34	30	-64	32
L PaCG	2640	8.48	-2	18	48
R SFG		7.61	28	4	64
R MFG		7.22	28	-4	52
R MFG	1972	7.7	50	30	30
R PreCG		7.06	46	2	30
R PreCG		6.91	54	10	34

L IPS/SMG	2081	7.52	-46	-40	48
L SPL/IPS		6.84	-28	-52	44
L IPS		6.38	-40	-54	54
L PreCG	695	6.99	-46	2	38
L Occipital Pole	942	6.7	-26	-94	-8
L LOC		5.54	-38	-82	-8
L Occipital Pole		3.73	-12	-100	-6
R AI	476	6.69	32	22	0
L AI	321	5.98	-32	24	-4
L ACC	361	4.92	-4	4	28
R ACC		4.26	6	2	28
R Caudate		4.00	12	-6	22
R Caudate	211	4.74	12	12	10
R Frontal Pole	207	4.45	48	44	-6
R Frontal Pole		2.89	34	56	-6
L SFG/Premotor	186	4.25	-24	-4	56
Med > Far					
R Frontal Pole	5112	6.61	44	38	22
R AI		6.20	32	22	6
L Putamen		5.25	-22	-2	18
R IPS/SPL	10285	6.57	36	-52	50
R LOC/IPS		6.53	32	-64	50
R LOC		5.41	32	-90	-2
R SFG/Premotor	1267	5.10	4	20	52
R ACC		4.94	8	18	32
R PaCG		4.80	8	24	42
R LG	262	4.95	26	-62	2
R LG		3.93	24	-48	-2
L LG	310	4.91	-24	-60	0
L LG		4.08	-20	-70	0
L PreCG	549	4.90	-46	2	34

L PreCG		4.36	-54	6	42
L Premotor/PreCG		3.50	-52	-2	50
L FOper	217	4.64	-30	22	10
R Thalamus	166	4.20	28	-32	2
R Lateral geniculate body		2.79	28	-24	-4
L Thalamus	213	4.11	-18	-34	0
L Lateral geniculate body		3.18	-24	-28	-4
L MFG	129	4.07	-46	32	36
L MFG		3.10	-42	22	30
L LOC/ITG	185	4.07	-40	-64	-6
L Frontal Pole	157	3.73	-44	44	2
L Frontal Pole		2.97	-38	46	12
R Occipital Pole	76	3.64	6	-96	16
R PreCG	232	3.35	2	-32	72

Abbreviations: ACC, Anterior Cingulate Cortex; AI, Anterior Insula; FOper, Frontal Operculum; HIPP, Hippocampus; ITG, Inferior Temporal Gyrus; IPS, Intraparietal Sulcus; LG, Lingual Gyrus; LOC, Lateral Occipital Cortex; MFG, Middle Frontal Gyrus; PaCG, Paracingulate Gyrus; PreCG, Precentral Gyrus; SFG, Superior Frontal Gyrus; SMG, Supramarginal Gyrus; SPL, Superior Parietal Lobule; L, Left; R, Right.

Table S10. Brain regions showing significant distance effects across all notations within each grade group.

			MNI coordinates		
**Region**	**Cluster Size**	**T-value**	**x**	**y**	**z**
2nd graders: Near > Far					
R SMG/IPS	847	5.14	46	-40	50
R LOC/IPS		4.36	26	-58	40
R LOC/IPS		4.19	26	-62	48
R FP/MFG	151	3.37	46	36	32
R FP/MFG		3.31	48	36	20
R FP		3.00	40	46	10
5th graders: Near > Far					
R SPL/IPS	3119	10.45	40	-46	50
R LOC/IPS		8.87	32	-58	46
R LOC/IPS		6.4	30	-66	34
R PaCG	6796	9.07	2	20	48
R IFG		9.01	34	20	4
R SFG		8.69	30	4	64
R Cerebellum	114	3.71	4	-60	-22
L AI	608	7.56	-30	22	2
L AI		4.13	-28	16	16
L SPL/IPS	2975	7.02	-42	-42	48
L LOC		5.98	-26	-72	28

L IPS		5.94	-36	-46	44
L PreCG	82	6.23	-38	0	30
L PreCG		3.9	-30	2	32
L PreCG	128	6.05	-48	0	40
L Thalamus	718	4.98	-16	-6	14
L Caudate		4.73	-12	10	0
L Thalamus		4.12	-10	-20	12
L MFG	214	4.63	-50	6	46
L MFG		4.59	-54	10	40
L MFG		4.19	-48	26	36
L FP	179	4.19	-44	42	2

Abbreviations: AI, Anterior Insula; FP, Frontal Pole; IFG, Inferior Frontal Gyrus; IPS, Intraparietal Sulcus; LOC, Lateral Occipital Cortex; MFG, Middle Frontal Gyrus; PaCG, Paracingulate Gyrus; PreCG, Precentral Gyrus; SFG, Superior Frontal Gyrus; SMG, Supramarginal Gyrus; SPL, Superior Parietal Lobule; L, Left; R, Right.

Table S11. Brain regions showing significant distance effects for each notation in 2nd-graders.

Region	Cluster Size	T-value	MNI coordinates x	y	z
Nonsym: Near > Far					
R AI	91	4.22	32	20	10
R LOC/IPS	215	3.86	26	-60	38
R MFG	74	3.58	48	34	28
R FP		2.86	42	38	40
R SMG/IPS	120	3.56	44	-38	44
Mixed: Near > Far					
R LOC	76	3.19	28	-70	32
Sym: Near > Far					
No significant clusters.					

Abbreviations: AI, Anterior Insula; FP, Frontal Pole; IPS, Intraparietal Sulcus; LOC, Lateral Occipital Cortex; MFG, Middle Frontal Gyrus; SMG, Supramarginal Gyrus; L, Left; R, Right.

Table S12. Brain regions showing significant distance effects for each notation in 5th-graders.

			MNI coordinates		
**Region**	**Cluster Size**	**T-value**	**x**	**y**	**z**
Nonsym: Near > Far					
R LOC/IPS	5555	7.24	22	-64	52
R SMG/IPS		6.79	46	-36	50
R PreCG		6.42	52	8	40
R PaCG	154	4.83	2	16	50
R SFG		3.13	0	28	50
R Caudate	389	4.61	14	18	6
R Caudate		4.09	14	0	14
R Thalamus		3.17	10	-14	12
L LOC	3549	5.74	-36	-82	-12
L SPL/IPS		5.71	-40	-44	52
L LOC		5.66	-30	-88	0
L Caudate	180	4.43	-10	8	8
L Putamen		2.83	-20	2	14
L FOper/AI	229	3.86	-32	26	6
L AI		3.74	-34	22	-6
L FOper		3.11	-32	14	16
L ACC	75	3.64	-6	4	26
L ACC		3.40	2	2	24
Mixed: Near > Far					

R SPL/IPS	1711	8.30	38	-42	42
R LOC/IPS		4.43	30	-66	36
R SFG	2224	6.10	26	-2	56
R SFG		5.26	28	4	64
L PaCG		5.06	-4	18	50
R MFG	1746	5.77	46	28	26
R PreCG		5.51	48	6	38
R FP		4.06	46	40	2
R AI	287	4.53	36	22	0
L SPL/IPS	1215	5.34	-42	-44	48
L IPS		4.52	-30	-50	42
L LOC/IPS		4.49	-26	-70	32
L PreCG	73	5.05	-38	2	26
L PreCG	138	4.85	-52	2	38
L PreCG		4.53	-50	2	46
L PreCG		2.96	-46	-2	54
L SFG	255	4.44	-24	-8	58
L Premotor		3.63	-32	-8	62
L SFG		3.61	-24	4	62
L AI	122	4.18	-32	20	0
L PCC	79	3.41	-2	-20	28
L PCC		3.31	-6	-34	24
Sym: Near > Far					

R IPS	258	4.42	40	-50	46
R AI	229	4.14	32	24	8
R FOper		3.28	42	18	0
R PreCG	146	3.98	44	8	30
R MFG	106	3.70	50	28	30
R FP		3.64	48	36	28
R MFG		2.96	40	28	26
R SFG	218	3.44	0	6	56
R PaCG		3.38	0	22	48
L PaCG		3.26	-4	26	42
L SMG/IPS	87	3.51	-44	-42	42
L LOC	71	3.49	-28	-68	40
L FOper/AI	169	4.08	-32	16	8
L AI		3.43	-32	22	-2
L FOper		3.35	-28	16	16

Abbreviations: AI, Anterior Insula; FOper, Frontal Operculum; FP, Frontal Pole; IFG, Inferior Frontal Gyrus; IPS, Intraparietal Sulcus; LOC, Lateral Occipital Cortex; MFG, Middle Frontal Gyrus; OFG, Occipital Fusiform Gyrus; PaCG, Paracingulate Gyrus; PCC, Posterior Cingulate Cortex; PreCG, Precentral Gyrus; SMG, Supramarginal Gyrus; SFG, Superior Frontal Gyrus; SPL, Superior Parietal Lobule; L, Left; R, Right.

Table S13. Brain regions showing significant correlation between neural distance effects for symbolic fractions and the degree of congruence, measured by reaction times (incongruent – congruent).

			**MNI coordinates**		
**Region**	**Cluster Size**	**T-value**	**x**	**y**	**z**
L ACC	87	3.71	-16	26	20
L ACC		3.31	-10	12	22
L LOC	77	3.55	-36	-72	30
L PaCG	75	3.43	-10	42	16
L FP	172	3.43	-6	60	18
R SFG/FP		3.22	4	56	20
L FP		2.97	-12	58	8

Abbreviations: ACC, Anterior Cingulate Cortex; FP, Frontal Pole; LOC, Lateral Occipital Cortex; PaCG, Paracingulate Gyrus; SFG, Superior Frontal Gyrus; L, Left; R, Right.

Table S14. Brain regions showing significant interactions between grade and distance.

			MNI coordinates		
**Region**	**Cluster Size**	**T-value**	**x**	**y**	**z**
5th graders (Near – Far) > 2nd graders (Near – Far)					
R MFG	1010	4.84	40	18	34
R PreCG		3.81	50	0	28
R IFG		3.71	36	16	22
R IPS	197	3.92	38	-44	44
R Premotor	116	3.47	28	-8	54
R Premotor		2.75	28	-6	42
R LG	138	3.45	2	-84	-10
R LG		3.09	12	-82	-4
R AI	142	3.17	36	24	-2
R FOper		3.10	46	18	-4
R IFG		2.92	38	34	2
L Caudate	2079	4.55	-20	-8	16
L Caudate		3.96	-14	10	6
L ACC		3.79	-8	2	30
L MFG	335	4.09	-54	22	32
L MFG		3.76	-54	10	40
L MFG		3.69	-46	28	36
L SMG	103	3.92	-58	-32	50

L LOC	112	3.51	-30	-90	-10
L LOC		3.2	-38	-82	-14
L IFG	187	3.39	-38	30	0
L FOper/AI		3.05	-42	20	-2
L FP		2.98	-42	46	6
2nd graders (Near – Far) > 5th graders (Near – Far)					
No significant clusters.					

Abbreviations: ACC, Anterior Cingulate Cortex; AI, Anterior Insula; FOper, Frontal Operculum; FP, Frontal Pole; IFG, Inferior Frontal Gyrus; IPS, Intraparietal Sulcus; LG, Lingual Gyrus; LOC, Lateral Occipital Cortex; MFG, Middle Frontal Gyrus; PreCG, Precentral Gyrus; SMG, Supramarginal Gyrus; SPL, Superior Parietal Lobule; L, Left; R, Right.

Table S15. Brain regions showing significant interactions between grade and distance within each notation.

Region	Cluster Size	T-value	MNI coordinates x	y	z
Nonsym: 5th graders (Near – Far) > 2nd graders (Near – Far)					
R MFG	255	4.15	42	16	34
R MFG		2.86	50	28	26
L MFG	179	5.28	-54	20	32
L LOC	200	3.70	-38	-82	-14
L Occipital Pole		3.63	-24	-92	-4
L LOC		3.62	-30	-90	-10
Mixed: 5th graders (Near – Far) > 2nd graders (Near – Far)					
R Premotor	454	3.86	28	-8	56
R MFG		3.66	30	0	58
R SFG		3.56	28	6	66
R PreCG	523	3.65	50	2	28
R MFG		3.53	46	10	36
R MFG		3.25	46	22	24
L Premotor	240	3.54	-28	-2	62
L SFG		3.46	-24	-8	58
L SFG		3.45	-6	12	56
L Thalamus	129	3.34	-20	-20	18
L Caudate		3.04	-20	-10	18

L Putamen		2.96	-16	-2	12
L ACC	71	3.1	0	10	40
Sym: 5th graders (Near – Far) > 2nd graders (Near – Far)					
L AI	90	3.81	-26	16	16
L AI		2.98	-30	14	8
L Pallidum	172	3.74	-20	-2	2
L Accumbens		3.41	-12	8	-6
L Caudate		3.36	-20	-6	22
L OFC	130	3.42	-22	24	-14
L AI		3.09	-32	24	-4

Abbreviations: ACC, Anterior Cingulate Cortex; AI, Anterior Insula; LOC, Lateral Occipital Cortex; MFG, Middle Frontal Gyrus; OFC, Orbitofrontal Cortex; PreCG, Precentral Gyrus; SFG, Superior Frontal Gyrus; L, Left; R, Right.

References

Bates, D., Mächler, M., Bolker, B., & Walker, S. (2014). Fitting linear mixed-effects models using lme4. *arXiv preprint arXiv:1406.5823*.

Fazio, L. K., DeWolf, M., & Siegler, R. S. (2016). Strategy use and strategy choice in fraction magnitude comparison. *Journal of Experimental Psychology: Learning, Memory, and Cognition*, *42*(1), 1.

Green, A. E., Kraemer, D. J. M., Fugelsang, J. A., Gray, J. R., & Dunbar, K. N. (2009). Connecting Long Distance: Semantic Distance in Analogical Reasoning Modulates Frontopolar Cortex Activity. *Cerebral cortex*, *20*(1), 70-76. https://doi.org/10.1093/cercor/bhp081

Hartogsveld, B., Bramson, B., Vijayakumar, S., van Campen, A. D., Marques, J. P., Roelofs, K., Toni, I., Bekkering, H., & Mars, R. B. (2018). Lateral frontal pole and relational processing: activation patterns and connectivity profile. *Behavioural Brain Research*, *355*, 2-11.

Holyoak, K. J., & Lu, H. (2021). Emergence of relational reasoning. *Current Opinion in Behavioral Sciences*, *37*, 118-124. https://doi.org/https://doi.org/10.1016/j.cobeha.2020.11.012

Kalra, P. B., Binzak, J. V., Matthews, P. G., & Hubbard, E. M. (2020). Symbolic fractions elicit an analog magnitude representation in school-age children. *Journal of experimental child psychology*, *195*, 104844. https://doi.org/10.1016/j.jecp.2020.104844

Mansouri, F. A., Koechlin, E., Rosa, M. G. P., & Buckley, M. J. (2017). Managing competing goals — a key role for the frontopolar cortex. *Nature reviews neuroscience*, *18*(11), 645-657. https://doi.org/10.1038/nrn.2017.111

Morales, N., Dartnell, P., & Gómez, D. M. (2020). A Study on Congruency Effects and Numerical Distance in Fraction Comparison by Expert Undergraduate Students [Original Research]. *Frontiers in Psychology*, *11*. https://doi.org/10.3389/fpsyg.2020.01190

Obersteiner, A., Van Dooren, W., Van Hoof, J., & Verschaffel, L. (2013). The natural number bias and magnitude representation in fraction comparison by expert mathematicians. *Learning and Instruction*, *28*, 64-72.

Schneider, M., & Siegler, R. S. (2010). Representations of the magnitudes of fractions. *Journal of Experimental Psychology: Human Perception and Performance*, *36*(5), 1227. https://doi.org/10.1037/a0018170
